# Silencing or inhibition of H3K79 methyltransferase DOT1L induces cell cycle arrest by epigenetically modulating c-Myc expression in colorectal cancer

**DOI:** 10.1186/s13148-019-0778-y

**Published:** 2019-12-30

**Authors:** Liqun Yang, Qian Lei, Lin Li, Jie Yang, Zhen Dong, Hongjuan Cui

**Affiliations:** 1 State Key Laboratory of Silkworm Genome Biology, Institute of Sericulture and Systems Biology, Southwest University, No.2, Tiansheng Road, Beibei, Chongqing, 400716 China; 2 Cancer Center, Medical Research Institute, Southwest University, Beibei, Chongqing, 400716 China; 3 Engineering Research Center for Cancer Biomedical and Translational Medicine, Southwest University, Beibei, Chongqing, 400716 China; 4 Chongqing Engineering and Technology Research Center for Silk Biomaterials and Regenerative Medicine, Southwest University, Beibei, Chongqing, 400716 China

**Keywords:** DOT1L, H3K79 methylation, Colorectal cancer, Epigenetics, c-Myc

## Abstract

**Background:**

Epigenetic regulations play pivotal roles in tumorigenesis and cancer development. Disruptor of telomeric silencing-1-like (DOT1L), also known as KMT4, is the only identified histone methyltransferase that catalyzes the mono-, di-, and tri-methylation of lysine 79 histone 3 (H3K79). However, little is known about the effect of H3K79 methylation on the modulation of colorectal cancer (CRC) development.

**Methods:**

DOT1L expression profiles in different subgroups of CRC tissues and its clinical significances were analyzed from some online datasheets. DOT1L in CRC cell lines was silenced by either lentivirus-mediated knockdown or inhibited by its specific inhibitor, EPZ004777. Then cell proliferation was detected by MTT assay, BrdU assay, and soft agar assay; cell cycle was detected by cytometry; and tumorigenicity was detected by using nude mice xenograft models. Clinical co-expression was analyzed between DOT1L and c-Myc. Chromatin immunoprecipitation (ChIP) assay was used to determine whether the translation of c-Myc was epigenetically regulated by H3K79me2 induced by DOT1L. c-Myc overexpression was used to rescue the cell cycle arrest and tumor growth induced by DOT1L silencing or inhibition in CRC.

**Results:**

We found that DOT1L was highly expressed in colorectal cancer and was negatively related to the prognosis of patients with CRC. Silencing or inhibition of DOT1L blocked cell proliferation, BrdU incorporation, self-renewal capability in vitro, and tumorigenicity in vivo. Besides, inhibition or silencing of DOT1L also induced cell cycle arrest at S phase, as well as decreased the expression of CDK2 and Cyclin A2. Furthermore, in the clinical databases of CRC, we found that the expression of DOT1L was positively correlated with that of c-Myc, a major regulator in the upstream of cell cycle–related factors. Besides, c-Myc expression was downregulated after DOT1L knockdown and c-Myc restoration rescued decrease of cell proliferation, BrdU corporation, self-renewal capability, cell cycle progression in vitro and tumorigenicity in vivo induced by DOT1L silencing. Then we found that H3K79 methylation was decreased after DOT1L knockdown. ChIP assay showed that H3K79me2 was enriched on the – 682~+ 284 region of c-Myc promoter, and the enrichment was decreased after DOT1L inhibition.

**Conclusions:**

Our results show that DOT1L epigenetically promotes the transcription of c-Myc via H3K79me2. DOT1L silencing or inhibition induces cell cycle arrest at S phase. DOT1L is a potential marker for colorectal cancer and EPZ004777 may be a potential drug for the treatment of colorectal cancer.

## Introduction

Colorectal cancer (CRC) is the third most frequent cancer and the fourth most frequent cancer cause of death in this world, accounting for roughly 1.4 million newly CRC-related patients and 0.7 million deaths each year [1, 2]. In China, colorectal cancer is similarly one of the five commonly diagnosed cancers, with a calculated 370,000 newly CRC-related patients and 191,000 deaths occurring in 2015 [3]. The morbidity and mortality are growing very fast in recent years. Therefore, searching for an effective therapeutic approach is urgent but cannot be achieved without understanding the fundamental research underlying the initiation and progression of colorectal cancer.

An increasing number of researchers pay attention to the post-translational histone modification due to their close connections with gene transcription, metabolism modulation, and cancer development [4–7]. Disruptor of telomeric silencing-1-like (DOT1L), a class I-like S-adenosyl-L-methionine (SAM)-binding methyltransferase, also called KMT4, is a conserved protein in mammals and is the only known histone methyltransferase that catalyzes the mono-, di-, and tri-methylation of H3K79 [8]. In addition, DOT1L is the only known histone methyltransferase lacking a SET domain and is regarded as an active transcription marker [9]. It plays important roles in different biological processes such as transcription elongation, cell cycle regulation, and DNA damage response [10–12].

Importantly, DOT1L also plays a pivotal role in tumors, especially leukemia [13, 14] and thymic lymphoma [15, 16]. In leukemia, DOT1L forms a protein complex with the mix lineage leukemia (MLL) fusion proteins and mediates H3K79 methylation, which is responsible for maintaining an open chromatin state around MLL fusion protein to target oncogenes [17–19]. Besides, DOT1L act as an oncogene by activating BAT1 and estrogen receptor α (ERα) to promotes migration and sphere formation of breast cancer [20, 21]. DOT1L also forms a novel transcriptional active complex with c-Myc and p300 to enhance epigenetic depression of epithelial-mesenchymal transition-related transcriptional factors (EMT-TFs) and promote EMT-induced cancer stem cell (CSC) properties in human breast cancer [22]. Besides, DOT1L is a novel co-factor in N-Myc-mediated transcriptional activation of target genes and neuroblastoma oncogenesis, and DOT1L inhibitors may be a clinical medication to treat N-Myc-amplified neuroblastoma [23]. DOT1L promotes miRNA-10b-mediated invasion and chemoresistance in head and neck squamous cell carcinoma (HNSCC) cancer stem cells [24]. However, in ovarian cancer, DOT1L inhibits cell invasion and cancer stem-like cell property [25]. In a recent study on pancreatic cancer and colon cancer, DOT1L is shown to epigenetically activated FOXM1, which inhibits maturation phenotypes and function of bone marrow–derived dendritic cells through the Wnt5a signaling pathway [26].

Importantly, DOT1L inhibitor EPZ5676 was studied in phase I clinical trials for the treatment of pediatric patients with relapsed/refractory leukemias bearing a rearrangement of the MLL gene (ClinicalTrials.gov Identifier: NCT02141828 and NCT01684150). However, its efficacy is modest as a single agent [18]. Another DOT1L inhibitor, EPZ004777 is a competitive antagonist of SAM, the coenzyme in the methylation [27, 28]. Previous reports had revealed the potential use of EPZ004777 as a promising drug for the treatment of acute myelocytic leukemia [29–32]. In colorectal cancer tissues, high DOT1L expression and H3K79me2 levels were associated with poor patient survival [33]. However, its mechanism has never been explored and little is known about the effect of EPZ004777 in this kind of solid tumor.

In this study, we aimed to explore the function of DOT1L, the effect of EPZ004777 and its molecular mechanism in colorectal cancer. We found that inhibition of DOT1L induces cell cycle arrest at S phase and suppresses cell proliferation in vitro and tumorigenicity in vivo. Our results provided an example of the relationship between histone methylation in colorectal cancer and DOT1L might be a potential therapeutic target for CRC treatment.

## Materials and methods

### Clinical data and samples

Clinical data were obtained from several online databases, such the Gene Expressing Profiling Interactive Analysis (GEPIA) [34, 35], the R2 platform [36], or the Oncomine [37]. Log-rank (Mentel-Cox) test was conducted for the significance in survival analysis. Scan cutoff modus was used to get the most significant expression cutoff in the Kaplan-Meier module for survival analysis. One-way analysis of variance (ANOVA) multiple comparison was performed for gene expression pattern in multiple groups.

### Cell culture and inhibitor treatment

All human colorectal cancer cell lines and 293FT were purchased from American Type Culture Collection (ATCC, Manassas, VA, USA). SW480 and SW620 cells were cultured in Dulbecco’s modified Eagle’s medium (DMEM), HCT116 was cultured in McCoy’s 5A Medium [38] and HCT15 was cultured in Roswell Park Memorial Institute-1640 (RPMI-1640), supplemented with 10% fetal bovine serum (FBS) and 1% penicillin and streptomycin (P/S). 293FT cells (ATCC) were also cultured in DMEM with 10% and 1% P/S, containing extra 0.5 mg/ml G418, 4 mM L-glutamine, 0.1 mM nonessential amino acids, and 1 mM sodium pyruvate. The 293FT transfection medium does not consist of penicillin and streptomycin and G418. All cell lines were tested mycoplasma negative and cultured in an incubator with 5% CO2 at 37 °C. The culture medium, fetal bovine serum, antibiotics, and supplements were purchased from Thermo Fisher. EPZ004777 was dissolved in dimethyl sulfoxide (DMSO) as 50 μM stock solutions.

### Vector construction, transfection, and infection

The RNAi target sites were designed and then synthesized by The Beijing Genomics Institute (BGI, Shenzhen, China) and were cloned into a lentiviral pLKO.1 vector. The sequences of shRNAs were listed as below:

shDOT1L-1-F: 5′-CCGGAACATCACTATGGCGTCGAGACTCGAGTCTCGACGCCATAGTGATGTTTTTTTG-3′;

shDOT1L-1-R: 5′-AATTCAAAAAAACATCACTATGGCGTCGAGACTCGAGTCTCGACGCCATAGTGATGTT-3′; shDOT1L-2-F: 5′-CCGGCACGTTGAACAAGTGCATTTACTCGAGTAAATGCACTTGTTCAACGTGTTTTTG-3′

shDOT1L-2-R: 5′-AATTCAAAAACACGTTGAACAAGTGCATTTACTCGAGTAAATGCACTTGTTCAACGTG-3′

Human full-length Myc (GenBank: V00568.1) cDNA was obtained by using PCR and was cloned into lentiviral pCDH-CMV-MCS-EF1-copGFP vector. The primers used as below:

c-Myc-F-(EcoRI): CCGGAATTC ATGCCCCTCAACGTTAGCTTCA;

c-Myc-R-(BamHI): CGCGGATCC TTACGCACAAGAGTTCCGTAGC.

Letivirural vector construction, transfection, and infection were employed as previously described [39].

### Quantitative RT-PCR

RNA was extracted using Trizol (Takara, Dalian, China) according to the manufacturer’s protocol and real-time quantitative PCR was performed as previously reported [40]. Results were calculated by virtue of the ΔΔCt method with β-actin as a control. The primers were listed as below: GAPDH-F: 5′-AACGGATTTGGTCGTATTGGG-3′; GAPDH-R: 5′-CCTGGAAGATGGTGATGGGAT-3′; DOT1L-F: 5′-CGCTGCCGGTCTACGATAAA-3′; DOT1L-R: 5′-TCGATGGCACGGTTGTACTT-3′; CyclinA2-F: 5′-CCTCCTTGGAAAGCAAACAGT-3′; CyclinA2-R:5′-CAGGGCATCTTCACGCTCTAT-3′; GAPDH-F:5′-AACGGATTTGGTCGTATTGGG-3′; GAPDH-R: 5′-CCTGGAAGATGGTGATGGGAT-3′; CDK2-F: 5′-GGCATTCCTCTTCCCCTCA-3′; CDK2-R: 5′-GCTCTGGCTAGTCCAAAGTCTG-3′; c-Myc-F: 5′-ACAGCCCACTGGTCCTCAAG-3′; c-Myc-R: 5′-TCGGTTGTTGCTGATCTGTCTC-3′.

### Western blot

Western blot was performed as previously described [40]. The antibodies used were listed as below: DOT1L (ab64077, Abcam), CDK2 (2546, CST), Cyclin A2 (4656, CST), PCNA (13110, CST), GAPDH (51332, CST), c-Myc (5605, CST), H3K79me1 (ab2886, Abcam, Cambridge, MA, USA), H3K79me2 (ab3594, Abcam), H3K79me3 (ab2621, Abcam), and H3 (17168-1-AP, Proteintech, Wuhan, China). Gray ratio of each blot was analyzed by using the Image J ver. 1.46 software and protein/GAPDH or protein/H3 ratio was shown.

### BrdU assay

The BrdU assay was performed according to the previous description [41]. Briefly, cells were seeded in 24-well plates with different treatment. Thereafter, it was incubated with 10 μg/ml thymidine analog 5-bromo-2-deoxyuridine (Brdu, Sigma) for 30 min at room temperature, then the supernatant was discarded and washed three times with PBS and fixed with 4% paraformaldehyde for 10 min. Then cells were pre-treated with 2 M HCl for 20 min and permeabilized with 0.5% Triton X-100 for 10 min. Afterwards, cells were blocked with 10% goat serum for 1 h, and then anti-Brdu monoclonal rat primary antibody (1:300, Sigma) was incubated overnight and incubated with 488 goat anti-rat IgG secondary antibody (H+L; Invitrogen). A total of 500 μl of DAPI was used for nuclear staining. Finally, the percentage of Brdu was calculated in more than 8 microscopic fields (Nikon 80i, Nikon Corporation, Tokyo, Japan).

### MTT assay

MTT assay was performed as previously described [42]. Briefly, 20 ul 5 mg/ml 3-(4,5-dimethyl-2-thiazolyl)-2,5-diphenyl-2-H-tetrazolium bromide (MTT, Sigma) was added into the growth medium in a well of 96-well plate with 1000 cells and was incubated for 4 h. After the precipitate was formed, the supernatant was carefully removed and was dissolved in 150 μl of DMSO, and the absorption value was measured at 560 nm using a MULTISCAN GO multilabel plate reader (Thermo).

### Cell cycle

Detection of cell cycle was according to previous report [40]. Briefly, cells were plated in 60-mm plates and performed with different treatment. Then cells were washed by pre-cooled PBS and anchored with 70% ethanol for more than 24 h. Thereafter, the cells were stained with propidium iodide (PI) and RNase for 1 h at 37 °C in the dark. Cells were finally collected by FACS C6 (BD Biosciences, San Jose, CA, USA) and analyzed using FlowJo 6.0 software.

### Soft agar assay

Colony formation ability was determined by soft agar assay on U87 cells by virtue of the method provided previously [43].

### Tumor xenografts

Four-week-old female mice (BALA/c-nu, Beijing Huafukang Bioscience Co. Inc., China) were purchased and housed in the IVC in a specific pathogen-free (SPF) room to acclimate for about a week. Then HCT116 and SW480 cells (1 × 10^6^) with gene alterations (including SW480-shGFP, SW480-shDOT1L-2, HCT116-shGFP, and HCT116-shDOT1L-2) in 100 μl PBS were subcutaneously injected into both flanks of the mice (shGFP in the left, while shDOT1L in the right, *N* = 3). Tumor growth was measured by caliper measurement every 2 days, and tumor volume was calculated after tumor plumped with the formula (volume = tumor length × width 2 × π/6). To explore the effect of EPZ004777 on xenografts of HCT116 cells in the BALB/c-nu nude mice, 1 × 10^6^ HCT116 cells were subcutaneously injected in the left flank of the BALB/c-nu mice (*N* = 6). After tumor pumped, the mice were randomly divided into two groups. One group was treated with PBS with 10% DMSO, while the other group was treated with EPZ004777 (100 mg/kg/day, diluted into PBS with 10% DMSO) for 16 days. At the termination of the experiment, tumors were removed and weighed.

### Hematoxylin-eosin staining and immunocytochemistry

Colorectal tissues (45) and normal colon tissues (*N* = 46) in a tissue microarray (CO1002b) were provided by US Biomax Inc. (Rockville, MD, USA) through the agency, the Alenabio Inc. (Xi’an, Shaanxi). Hematoxylin-eosin (H&E) and immunocytochemistry (IHC) were performed as previously described [41]. Antibodies used were the following: DOT1L (ab64077, Abcam), c-Myc (ab32072, Abcam), Ki67 (ab15580), H3K79me1 (ab2886, Abcam), H3K79me2 (ab3594, Abcam), and H3K79me3 (ab2621, Abcam). Signal-positive rate was calculated by using the IHC profiler in the Image J ver.1.46 software.

### Chromatin immunoprecipitation assay and CHIP-seq analysis

CHIP-seq data (GSE74812; BED files) of H3K79me2 and H3K79me3 in human t(4;11) cell line [44] was downloaded from GEO and promoter enrichment region was analyzed by using the IGV 2.6.3 software. Chromatin immunoprecipitation (ChIP) assay was performed in HCT116 and SW480 cells by using the EZ CHIP^TM^ kit as according to the manufacturer’s protocol. The sequences of c-Myc promoter were downloaded from the UCSC website [45]. Antibodies that targeted H3K79me1, H3K79me2, H3K79me3, and DOT1L were purchased from Abcam and normal rabbit IgG was obtained from Beyotime (Taicang, Jiangsu, China). Primers used were shown as below:

c-Myc~-2682~-1682-F: 5′-CCTCCAGTAACTCCTCTTTCTTCG-3′;

c-Myc~-2682~-1682-R: 5′-TCTTCTCATCCTTGGTCCCTCA-3′;

c-Myc~-1682~-682-F: 5′-GCAATGCGTTGCTGGGTT-3′;

c-Myc~-1682~-682-R: 5′-CGTTCAGAGCGTGGGATGTT-3′;

c-Myc~-682~+284-F: 5′-TGCCTCTATCATTCCTCCCTATC-3′;

c-Myc~-682~+284-R: 5′-TCGGGTGTTGTAAGTTCCAGTG-3′;

c-Myc~+284~+1284-F: 5′-TTCGGCTCACCGCATTTC-3′

c-Myc~+284~+1284-R: 5′-CAACACCACGTCCTAACACCTCT-3′;

c-Myc~+1284~+2284-F: 5′-AAGAAGAAAAGCTGGCAAAAGG-3′;

c-Myc~+184~+2284-R: 5′-CCAAAATCCAAGGCACAAAGT-3′.

### Statistical analysis

All experimental data analysis was performed in a software GraphPad Prism 8 (https://www.graphpad.com/). Unpaired two-tailed Student’s *t* test was used for statistical analysis between two groups. One-way analysis of variance (ANOVA) multiple comparison was performed for gene expression pattern in multiple clinical groups. Person correlation was used to analyze the correlations between the expressions of 2 genes. *P* < 0.05 was considered significant and was marked with * in the figures. *P* < 0.01 was marked with **. *P* < 0.001 was marked with ***.

## Results

### DOT1L is highly expressed in CRC

**Fig. 1:**
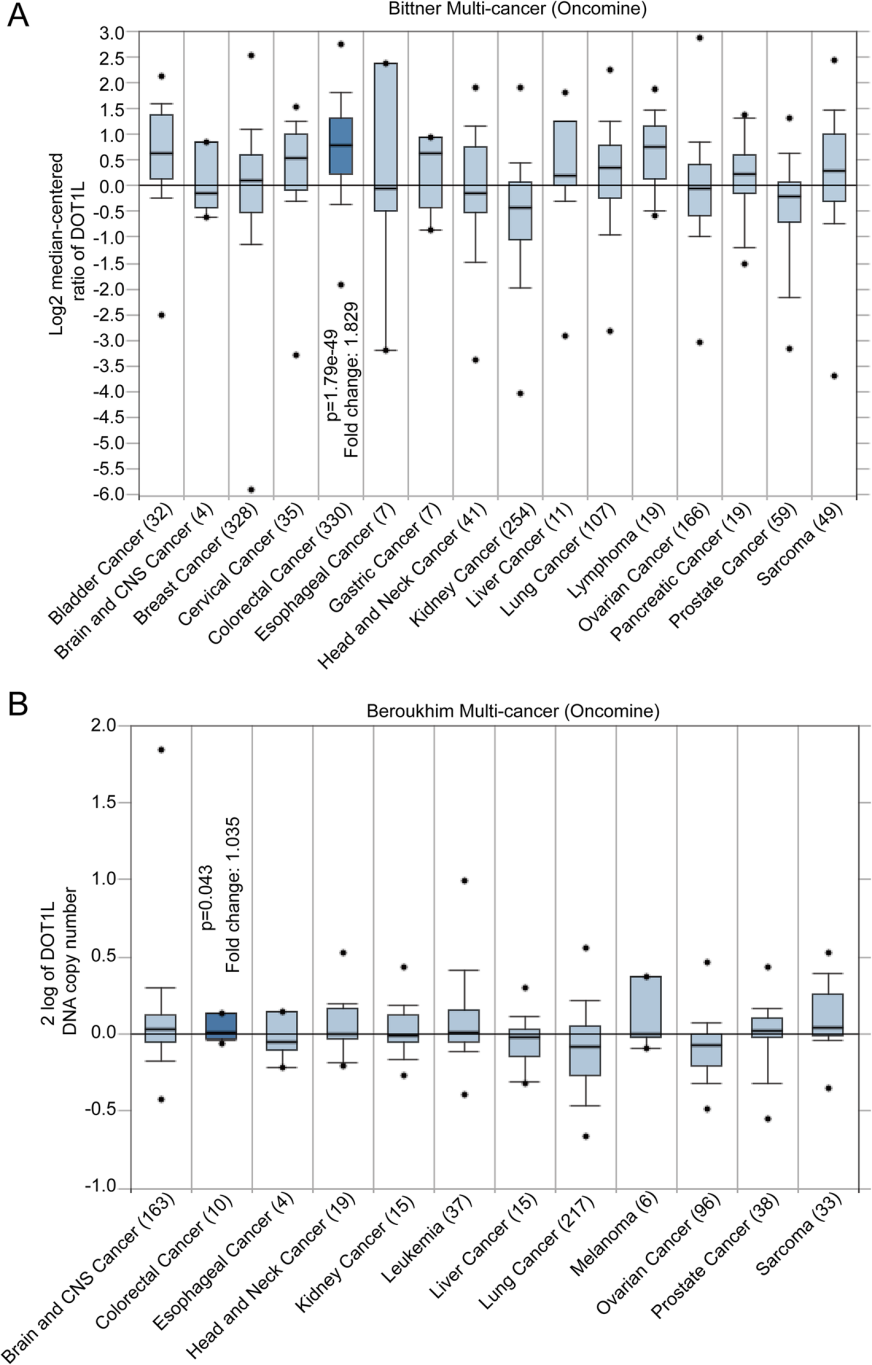
DOT1L is highly expressed in colorectal cancer than other cancer types. **a** The relative mRNA expression of DOT1L in multiple cancer types in Bittner Multi-cancer datasheet from the Oncomine. **b** DOT1L DNA copy number in multiple cancer types in Beroukhim multi-cancer datasheet from the Oncomine

**Fig. 2:**
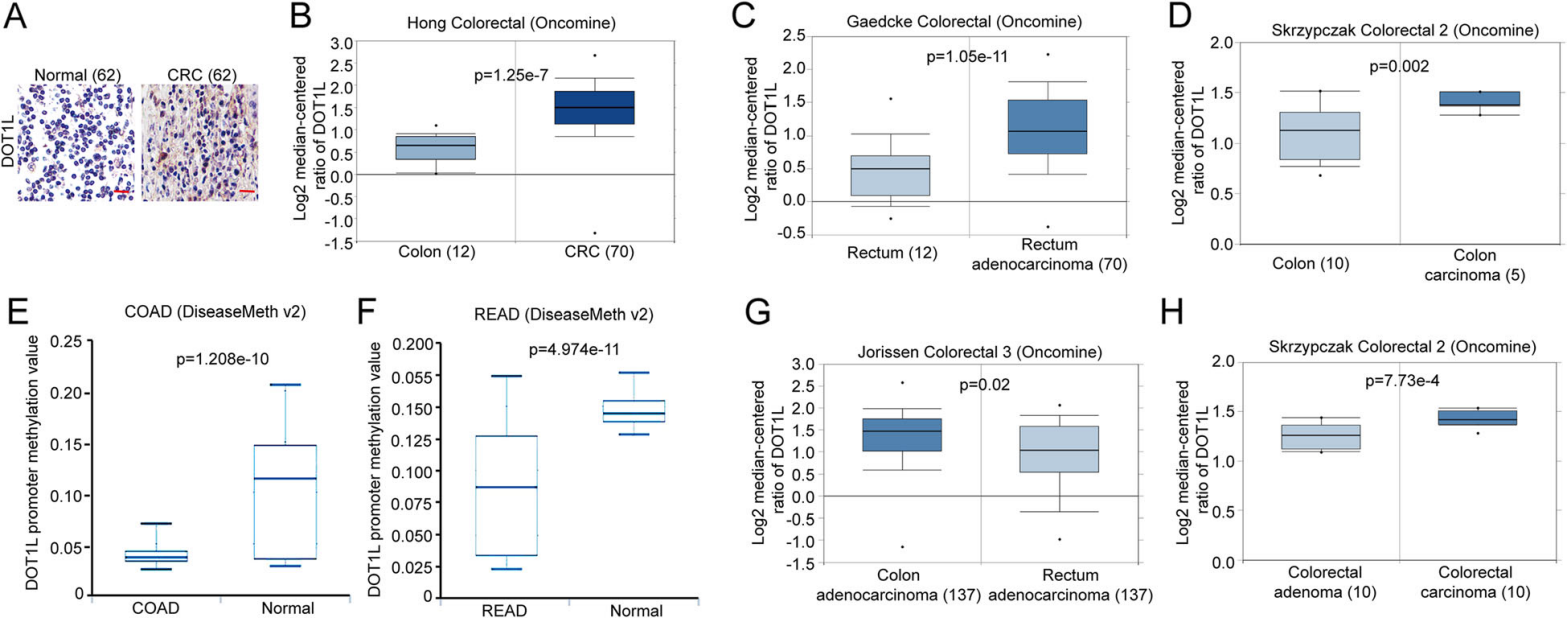
DOT1L is upregulated in colorectal cancer, especially in colorectal carcinoma. **a** Immunohistochemical (IHC) staining analysis of DOT1L expression in colorectal cancer (*N* = 45) tissues and normal tissues (*N* = 46). **b** Relative mRNA expression of DOT1L in normal colon or CRC tissues in Hong colorectal datasheet from the Oncomine. **c** Relative mRNA expression of DOT1L in normal rectum or rectum adenocarcinoma tissues in Gaedcke colorectal datasheet from the Oncomine. **d** Relative mRNA expression of DOT1L in normal colon or COAD tissues in Skrzypczak colorectal 2 datasheet from the Oncomine. **e** DOT1L promoter methylation in COAD or normal tissues from DiseaseMeth v.2. **f** DOT1L promoter methylation in READ or normal tissues from DiseaseMeth v.2. **g** Relative mRNA expression of DOT1L in COAD or READ tissues in Jorissen colorectal 3 datasheet from the Oncomine. **h** Relative mRNA expression of DOT1L in colorectal adenoma or colorectal carcinoma tissues in Skrzypczak colorectal 2 datasheet from the Oncomine

To reveal the function of DOT1L in CRC, we firstly compared the expression of DOT1L in CRC and other types of cancers in Bittner multi-cancer datasheet from the Oncomine. The result showed that DOT1L was highly expressed in CRC compared with other 15 types of cancers (Fig. 1a). Besides, we also found that the DNA copy number in both CRC tissues and CRC cell clines were relatively higher than other types of cancers (Fig. 1b and Additional file 1: Figure S1). Then we used IHC staining to detect the protein expression of DOT1L in CRC tissues and normal tissues, the result showed that DOT1L expression was higher than that of the normal tissues (Fig. 2a). By analyzing the expression of DOT1L in several different datasheets, we found that DOT1L expression or DNA copy number in colon carcinoma, rectum adenocarcinoma (READ), and colon adenocarcinoma (COAD) were higher than that of the carcinoma-associated fibroblasts, normal rectum, and colon, respectively (Fig. 2b–d and Additional file 1: Fig. S2 A–C). Actually, the methylation of DOT1L gene promoter in COAD or READ was lower than that of the normal tissues (Fig. 2e, f). CRC that is located in the proximal region (colon cancer) is commonly more malignant than that of the distal region (rectum cancer) [46]. Besides, in the development of colorectal cancer, adenoma is a pre-malignant lesion, while carcinoma is a malignant lesion [47]. Intriguingly, DOT1L expression in COAD was higher than that of READ (Fig. 2g), and was highly expressed in colorectal carcinoma than that of colorectal adenoma (Fig. 2h). Mucinous colorectal adenocarcinoma, a distinct subtype of CRC characterized by the presence of abundant extracellular mucin, is more frequently located in the proximal region and diagnosed at an advanced stage [48]. Interestingly, DOT1L expression in colorectal mucinous adenocarcinoma is the highest among several different types of CRCs, such as COAD, READ, and rectosigmoid adenocarcinoma (Additional file 1: Figure S2D). Consistently, DOT1L expression was also expressed higher in CRC that is located in the proximal region than that of the distal region (Additional file 1: Figure S2E). These results revealed that DOT1L is highly expressed in CRC, especially in COAD.

### DOT1L is correlated with poor prognosis of patients with CRC

**Fig. 3:**
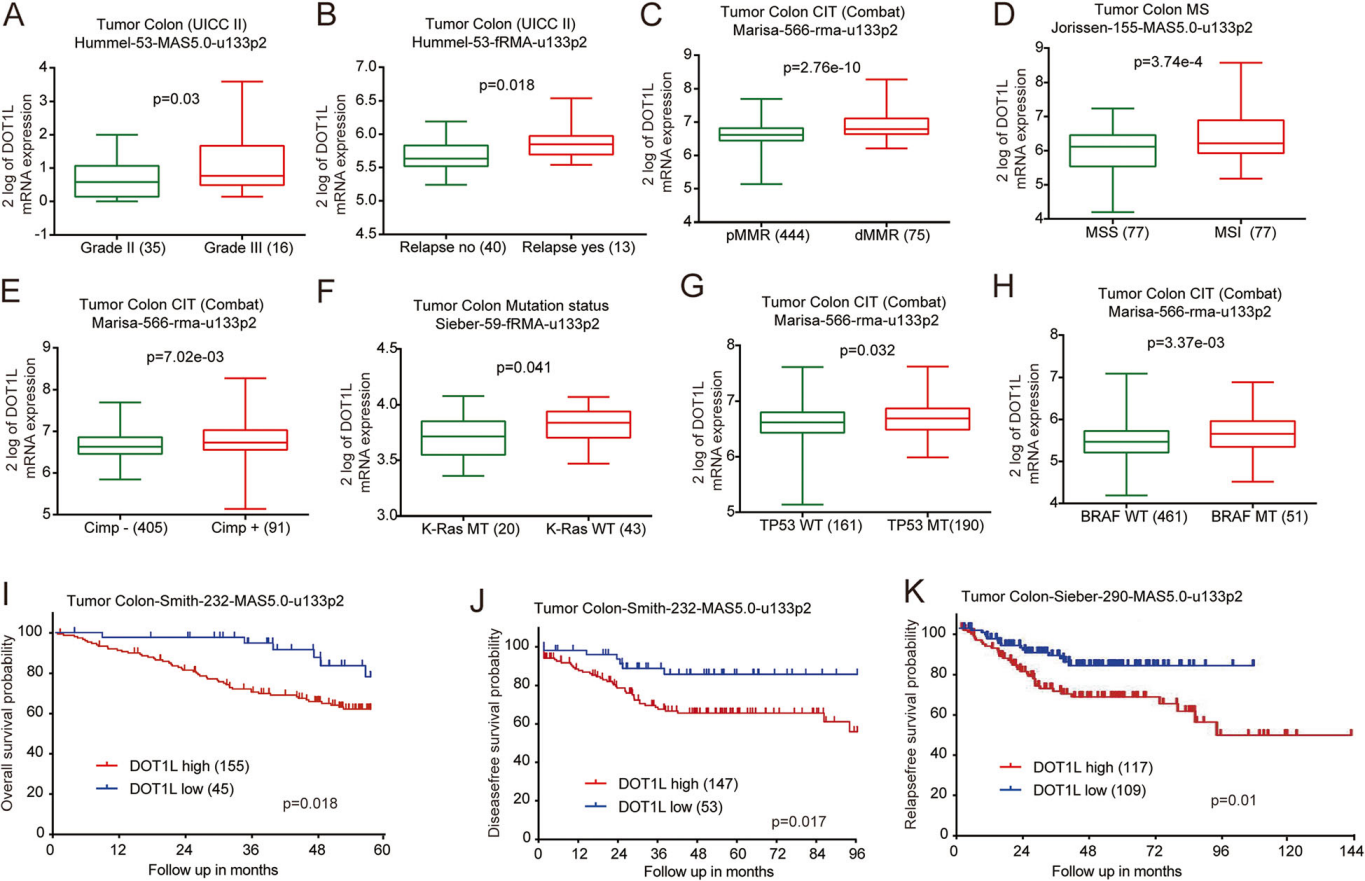
High expression of DOT1L predicts poor prognosis of CRCs. **a** Relative mRNA expression of DOT1L in grade II or III colon cancers in Hummel colon datasheet from the R2 platform. **b** Relative mRNA expression of DOT1L in colon cancers with relapse or not in Hummel colon datasheet from the R2 platform. **c** Relative mRNA expression of DOT1L in colon cancers with DNA mismatch repair-proficient (pMMR) or DNA mismatch repair-deficient (dMMR) in Marisa colon datasheet from the R2 platform. **d** DOT1L mRNA expression in colon cancers with microsatellites stability (MSS) or microsatellites instability (MSI) in Jorissen colon datasheet from the R2 platform. **e** DOT1L mRNA expression in colon cancers with high or low CpG island methylator phenotype (Cimp+/-) in Marisa colon datasheet from the R2 platform. **f** DOT1L mRNA expression in colon cancers with K-Ras mutation (MT) or wild-type (WT) in Sieber colon datasheet from the R2 platform. **g** DOT1L mRNA expression in colon cancers with TP53 mutation (MT) or wild-type (WT) in Marisa colon datasheet from the R2 platform. **h** DOT1L mRNA expression in colon cancers with BRAF mutation (MT) or wild-type (WT) in Marisa colon datasheet from the R2 platform. **i** Kaplan-Meier analysis of the relationship of DOT1L expression with overall survival (OS) probability in Smith colon cancer cohorts from the R2 platform. **j** Kaplan-Meier analysis of the relationship of DOT1L expression with disease-free survival (DFS) probability in Smith colon cancer cohorts from the R2 platform. **k** Kaplan-Meier analysis of the relationship of DOT1L expression with relapse-free survival (RFS) probability in Sieber colon cancer cohorts from the R2 platform

Furthermore, DOT1L expression was shown to be highly expressed in grade III colon cancer, compared to that of the grade II colon cancer (Fig. 3a). Besides, CRC patients with relapse had a higher DOT1L expression than that of CRC patients without relapse (Fig. 3b). CRC with DNA mismatch repair-deficient (dMMR) [49], high-microsatellites instability (MSI) [50], or high CpG island methylator phenotype (Cimp+) [51] have a poorer prognosis than that with DNA mismatch repair-proficient (pMMR), microsatellites stability (MSS), or low CpG island methylator phenotype (Cimp-). Interestingly, DOT1L was highly expressed in colon cancers with these characteristics (Fig. 3c–e and Additional file 1: Figure S3A–F). In addition, we also noted that DOT1L expression was also downregulated in colon cancers with K-Ras mutations (Fig. 3f), which predicted a better prognosis [52], and upregulated in colon cancers with TP53 and BRAF mutations (Fig. 3g, h and Additional file 1: Figure S3G), which predicted a poorer prognosis [53, 54]. Moreover, DOT1L expression was correlated with metastasis of colon cancers according to the analysis in several clinical datasheets (Additional file 1: Figure S3H–L). DOT1L was highly expressed in colon cancers with responder to FOLFOX6 (leucovorin, fluorouracil, oxaliplatin) treatment, compared with that without responder to FOLFOX6 treatment (Additional file 1: Figure S3M). Male patients with CRC commonly have a higher risk than that of the female patients [55]. Interestingly, DOT1L preferred to be highly expressed in colon cancer from male patients than that of the female patients (Additional file 1: Figure S3N–P). Black people seem to have the highest odds for colorectal cancer mortality than other races [56]. Colon cancer in Black people seemed to have a higher DOT1L expression than other races including White and Yellow people (Additional file 1: Figure S3Q). Then we analyzed the correlation of DOT1L expression and the prognosis of patients with colon cancers by using Kaplan-Meier (KM) analysis. The results showed that DOT1L expression was negatively correlated with the overall survive (OS) probability, disease-free survival (DFS) probability, and relapse-free survival (RFS) probability, respectively (Fig. 3i–k, Additional file 1: Figure S3R). These evidences suggested that DOT1L tends to be highly expressed in high-risk patients with CRC and predicts poor prognosis.

### DOT1L silencing or inhibition blocks cell proliferation of CRC cells in vitro

**Fig. 4:**
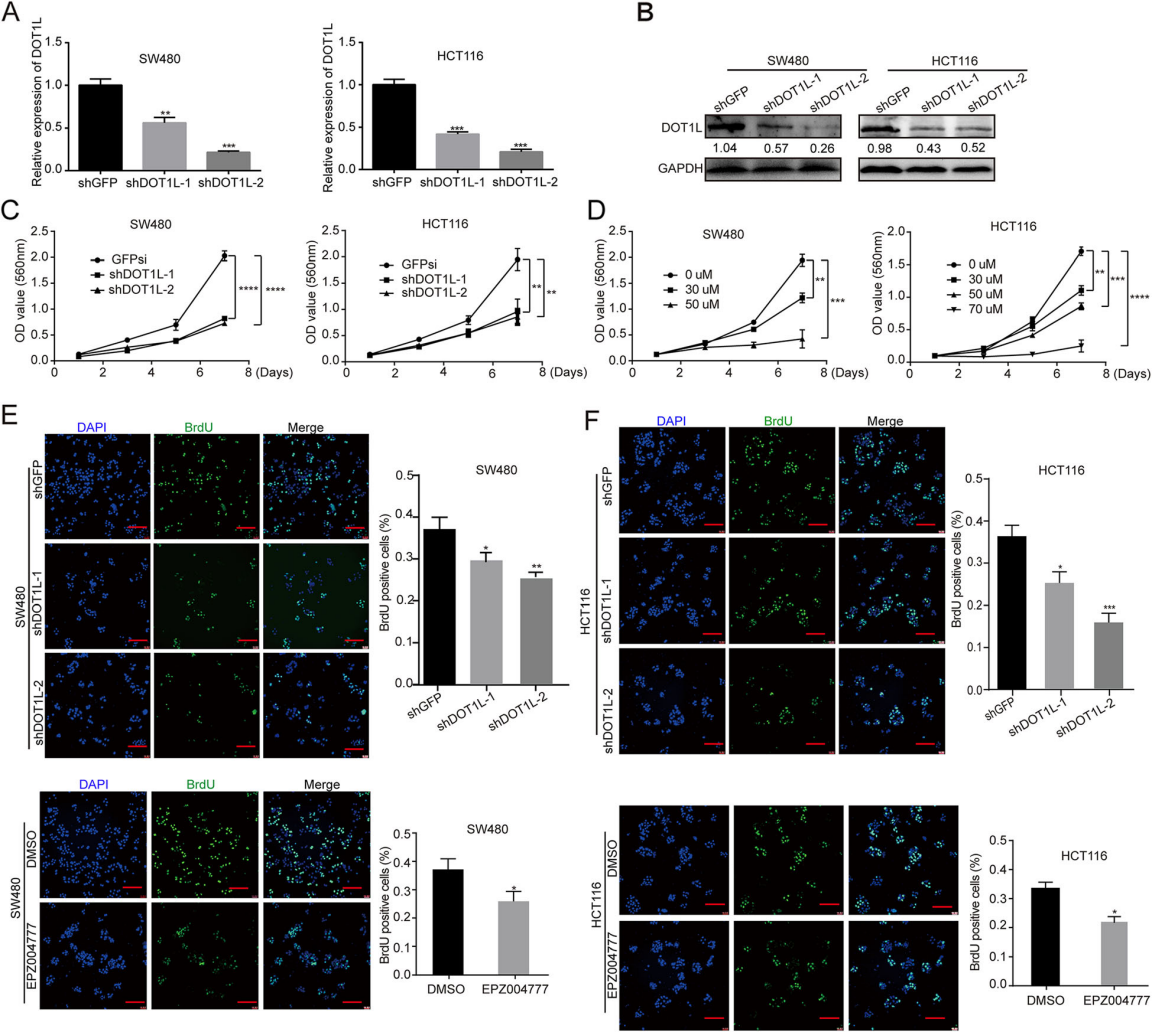
DOT1L silencing or inhibition blocks cell proliferation of CRC cells in vitro. **a** Relative mRNA expression of DOT1L detected by using qRT-PCR in SW480 and HCT116 colorectal cancer cell lines after DOT1L knockdown. shGFP vectors were used as control. **b** Protein expression of DOT1L detected by using Western blot in SW480 and HCT116 cells after DOT1L knockdown. Gray ratio of each blot was analyzed by using the Image J software and DOT1L/GAPDH ratio was shown. **c**, **d** Cell growth curve was determined by using MTT assay in SW480 and HCT116 cells after DOT1L knockdown or treatment with its specific inhibitor EPZ004777 with different concentrations for 1/3/5/7 days. **e, f** Cell proliferation was detected by using BrdU immunofluorescence in SW480 and HCT116 cells after DOT1L knockdown or inhibited by using EPZ004777 for 48 h (30 μM in SW480 and 50 μM in HCT116)

Next, we analyzed DOT1L mRNA and protein expression in several CRC cell lines including HCT15, SW620, SW480, and HCT116. The results showed that DOT1L was commonly expressed in these cell lines (Additional file 1: Figure S4A–B). To further elucidate the function of DOT1L in CRC cells, we silenced DOT1L expression in SW480 and HCT116 cells by using lentivirus-mediated shRNA stable transfection (Fig. 4a, b). Significantly, DOT1L silencing decreased cell proliferation in two cell lines, compared with an inactive GFP silencing (Fig. 4c). Besides, a specific DOT1L inhibitor EPZ004777 was used to inhibit the activity of DOT1L without affecting its expression (Additional file 1: Figure S4C, D). The results showed that 30 μM and 50 μM EPZ004777 significantly reduced cell viability of SW480 cells in a dose-dependent manner (Fig. 4d, left panel). However, HCT116 was less sensitive to EPZ004777 treatment. The cell viability was also inhibited by 30 μM, 50 μM, and 70 μM EPZ004777 treatment in a dose-dependent manner (Fig. 4f, right panel). Besides, DOT1L silencing or inhibition also reduced BrdU incorporation in SW40 and HCT116 cells (Fig. 4e, f). These results indicated that DOT1L silencing or inhibition blocked cell proliferation in CRC cells.

### DOT1L silencing or inhibition suppresses tumorigenicity of CRC cells in vivo

**Fig. 5:**
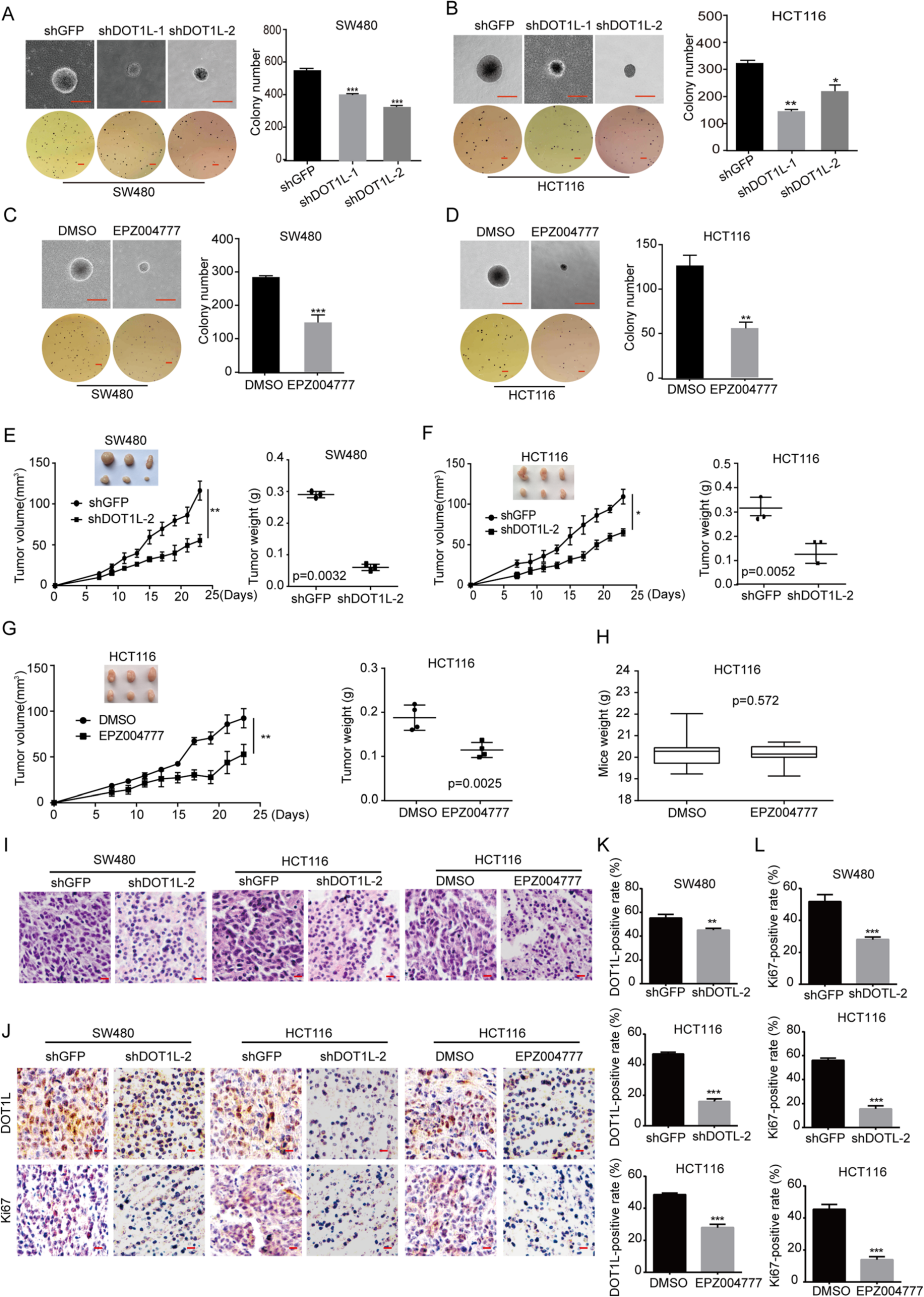
DOT1L silencing or inhibition suppresses tumorigenicity of CRC cells in vivo. **a–d** Self-renewal capacity detected by using soft agar assay in SW480 and HCT116 cells after DOT1L knockdown for 3 weeks or inhibited by using EPZ004777 for 2 weeks (30 μM in SW480 and 50 μM in HCT116). **e, f** The capacity of tumorigenicity was detected in BALB/c-nu mice subcutaneous injected with SW480 and HCT116 cells after DOT1L knockdown. Tumor volume was detected every 2 days from a week after subcutaneous injection. Tumor weight was measured after the tumors were removed from the bodies when the experiment was ended. **g, h** The capacity of tumorigenicity was detected in BALB/c-nu mice subcutaneous injected with HCT116 cells. After a week, mice were treated with EPZ004777 (100 mg/kg/day, diluted into PBS with 10% DMSO) or control solvent via intraperitoneal injection for 16 days. Tumor volume was detected every 2 days from the first time of intraperitoneal injection. Tumor weight and mice weight were measured after the tumors were removed from the bodies when the experiment was ended. **i** H&E staining of xenografts obtained from subcutaneous injecting SW480 and HCT116 cells after DOT1L knockdown or inhibition within mice. **j–l** IHC staining of DOT1L and Ki67 in xenografts obtained from subcutaneous injecting SW480 and HCT116 cells after DOT1L knockdown or inhibition within mice. Signal-positive rate was analyzed by using the IHC profiler in the Image J software

Furthermore, we confirmed the effect of DOT1L silencing or inhibition in CRC cells in vivo. Before that, we firstly tested the self-renewal capacity of CRC cells after DOT1L silencing or inhibition by virtue of soft agar experiment. Significantly, DOT1L silencing or inhibition reduced the size and number of colony formation in SW480 and HCT116 cells, compared with GFP silencing (Fig. 5a–d). Based on the results obtained above, we sought to explore the role of DOT1L in tumor progression in vivo by injecting 1 × 10^6^ SW480-shGFP, SW480-shDOT1L-2, HCT116-shGFP, and HCT116- shDOT1L-2 cells subcutaneously in both flanks of the BALB/c-nu mice (shGFP in the left, while shDOT1L in the right, *N* = 3). The results showed that the tumor volumes and weights of all DOT1L silenced tumors in the nude mice were significantly smaller or lighter than the control groups, respectively (Fig. 5e, f). Besides, we also explored the effect of EPZ004777 on xenografts of HCT116 cells in the BALB/c-nu nude mice. We injected 1 × 10^6^ HCT116 cells subcutaneously in the left flank of the BALB/c-nu mice (*N* = 6). After tumor pumped, we divided the mice into two groups randomly. One group was treated with PBS with 10% DMSO, while another group was treated with EPZ004777 (100 mg/kg/day, diluted into PBS with 10% DMSO) for 16 days. The results showed that the tumor volumes and weights of all EPZ004777-treated tumors in the nude mice were significantly smaller and lighter than the control groups, respectively (Fig. 5g). However, the weights of the mice between EPZ004777- and PBS-treated groups had no significance (Fig. 5h). Hematoxylin-eosin (H&E) staining revealed that nucleocytoplasmic ratio was decreased after DOT1L knockdown or inhibition (Fig. 5i). Immunostaining (IHC) analysis further confirmed the downregulation of DOT1L and Ki67 protein in DOT1L-silenced cells (Fig. 5j–l). Taken together, these results suggested that DOT1L was essential for the tumorigenicity of GBM cells in vivo and EPZ004777 might be a potential drug for CRC treatment.

### DOT1L silencing or inhibition induces cell cycle arrest in S phase

**Fig. 6:**
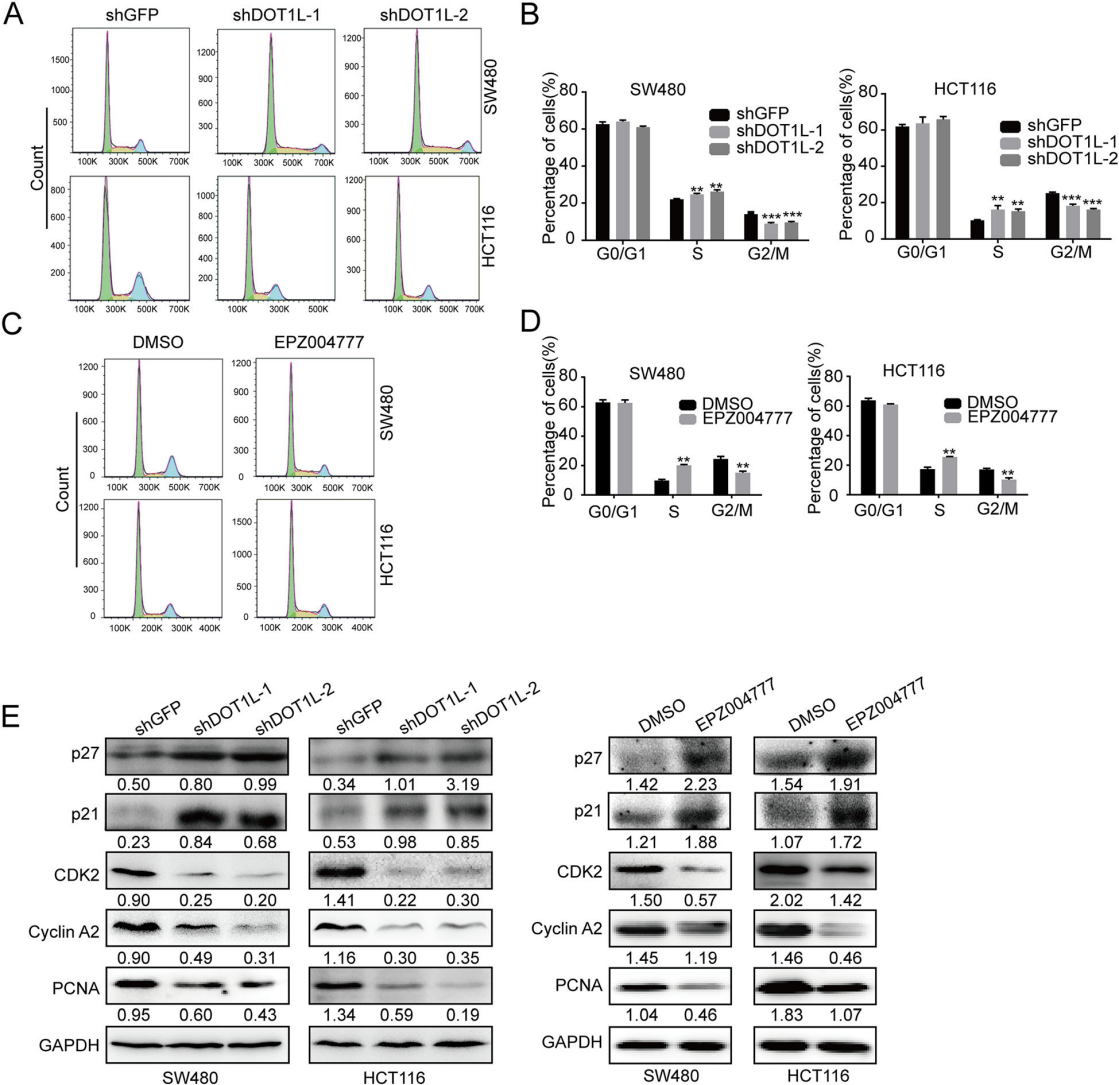
DOT1L silencing or inhibition induces cell cycle arrest in S phase. **a–d** Cell cycle detected by flow cytometry in SW480 and HCT116 cells after DOT1L knockdown or inhibition. Cells were stained by propidium iodide (PI) and RNase in 37 °C for 30 min before flow cytometry. **e** Protein expression of cell cycle–related proteins including p21, p27, CDK2, Cyclin A2, and PCNA was detected by using Western blot in SW480 and HCT116 cells after DOT1L knockdown or inhibition. Gray ratio of each blot was analyzed by using the Image J software and protein/GAPDH ratio was shown

Since cell cycle is important for cell proliferation, we subsequently study the effect of DOT1L silencing or inhibition on cell cycle in CRC cells. Flow cytometry analysis of PI staining showed that DOT1L silencing or inhibition induced cell cycle arrest at S phase in SW480 and HCT116 cells, compared with an inactive GFP silencing (Fig. 6a–d). Besides, we found that protein expression of cell cycle–related proteins such as CDK2, Cyclin A2, and PCNA were significantly reduced after DOT1L knockdown or inhibition, while cell cycle inhibitor p21 and p27 expression were significantly upregulated (Fig. 6e). These results showed that DOT1L silencing or inhibition probably inhibits cell proliferation via regulation of cell cycle in CRC cells.

### DOT1L silencing or inhibition demethylates H3K79 and suppresses transcription of c-Myc

**Fig. 7:**
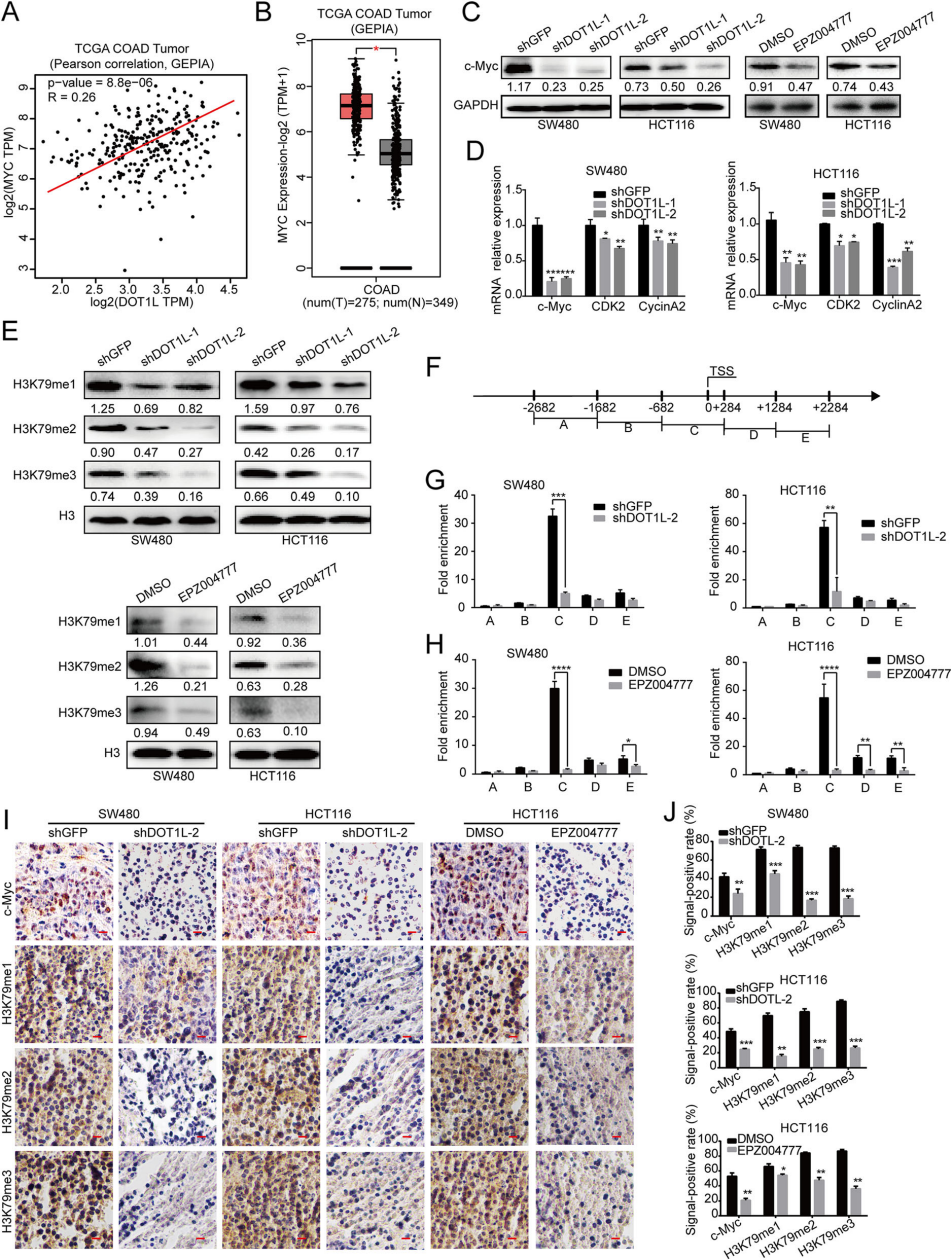
DOT1L silencing or inhibition demethylates H3K79 and suppresses transcription of c-Myc. **a** The Pearson correlation between DOT1L and c-Myc expression in patients with colorectal cancer in the TCGA COAd datasheet from the GEPIA. **b** Relative mRNA expression of c-Myc in patients with colorectal cancer in the TCGA COAd datasheet from the GEPIA. **c** Protein expression of c-Myc in SW480 and HCT116 cells after DOT1L knockdown or inhibition. Gray ratio of each blot was analyzed by using the Image J software and protein/GAPDH ratio was shown. **d** mRNA expression of c-Myc, CDK2, Cyclin A2 in SW480, and HCT116 cells after DOT1L knockdown or inhibition. **e** H3K9 methylation (m1/2/3) was detected by using Western blot in SW480 and HCT116 cells after DOT1L knockdown or inhibition. Gray ration of each blot was analyzed by using the Image J software and protein/H3 ratio was shown. **f–h** ChIP assay was performed to detect the binding region of H3K79me2 on the promoter of c-Myc in SW480 and HCT116 cells after DOT1L knockdown or inhibition. **i**, **j** IHC staining of c-Myc and H3K79me1/2/3 in xenografts obtained from subcutaneous injecting SW480 and HCT116 cells after DOT1L knockdown or inhibition within mice. Signal-positive rate was analyzed by using the IHC profiler in the Image J software

c-Myc is a oncogene contributing to cell cycle procession and tumor development. Interestingly, we found that DOT1L mRNA expression is positively correlated with MYC mRNA expression (Fig. 7a, Additional file 1: S5A, B). Besides, MYC is highly expressed in COAD, compared with that of normal colons (Fig. 7b). Therefore, we used RT-PCR and Western blot assays to find that both c-Myc RNA levels and protein levels were reduced after DOT1L silencing or inhibition (Fig. 7c, d). Besides, c-Myc was downregulated in xenografts of CRC cells with DOT1L silencing or inhibition (Fig. 7 i). Importantly, the mRNA expression of CDK2 and CCNA2, which are target genes of c-Myc, were also decreased after DOT1L silencing or inhibition (Fig. 7d). DOT1L can catalyze the mono-methylation, di-methylation, and tri-methylation of H3K79, and that H3K79me2 has been shown to activate the transcription of related genes [57]. As expected, H3K79 monomethylation, dimethylation, and trimethylation was all decreased after DOT1L silencing or inhibition (Fig. 7e). By analyzing CHIP-seq data, we found that H3K79me2/3 was enriched in the promoter region of c-Myc in human cells (Additional file 1: Figure S5C). In order to further explore the relationship between DOT1L and c-Myc, we designed related primers in the c-Myc promoter region and found that H3K79me2 was enriched on the − 6682~+ 284 region of c-Myc promoter (Fig. 7f–h). Besides, H3K79me1/2/3 levels were also significantly descreased after DOT1L silencing or inhibition in the xenografts of CRC cells in vivo experiments (Fig. 7 i, j). Therefore, our data revealed that DOT1L silencing might inhibit the transcriptional expression of c-Myc by inhibiting methylation of H3K79.

### Restoration of c-Myc partly rescued cell proliferation inhibition and cell cycle arrest induced by DOT1L silencing or inhibition in vitro and in vivo

**Fig. 8:**
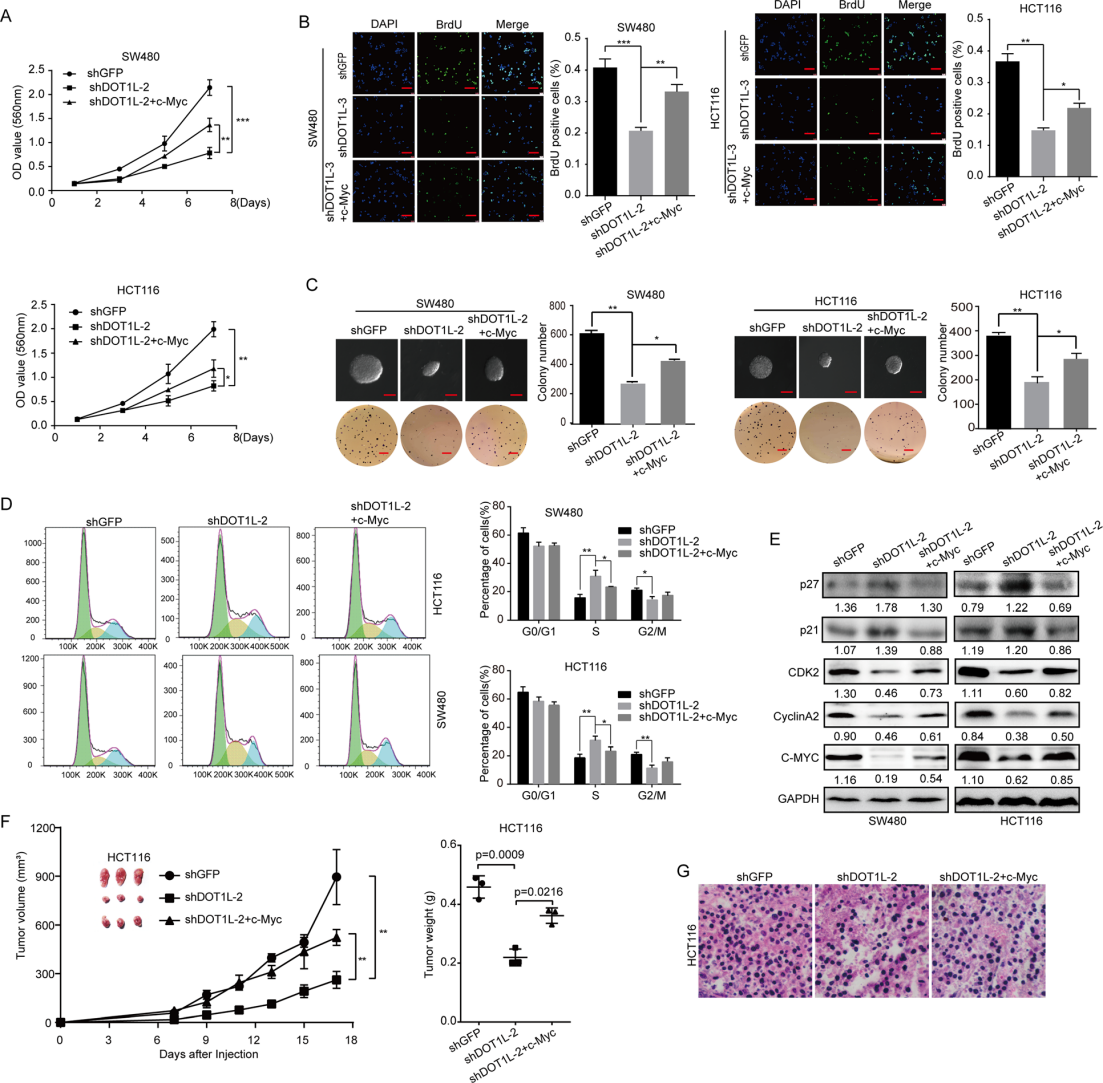
Restoration of c-Myc partly rescued cell proliferation inhibition and cell cycle arrest induced by DOT1L silencing or inhibition in vitro and in vivo. **a** Cell growth curve was determined by using MTT assay in SW480 and HCT116 cells after DOT1L knockdown and c-Myc restoration. Vector control for c-Myc overexpression was used in both shGFP and shDOT1L-2 groups (similarly hereinafter). **b** BrdU assay was performed in SW480 and HCT116 cells after DOT1L knockdown and c-Myc restoration. **c** Soft agar assay was performed in SW480 and HCT116 cells after DOT1L knockdown and c-Myc restoration. **d** Cell cycle was detected by using flow cytometry in SW480 and HCT116 cells after DOT1L knockdown and c-Myc restoration. **e** Protein expression of cell cycle–related proteins including p21, p27, CDK2, Cyclin A2, and PCNA was detected by using Western blot in SW480 and HCT116 cells after DOT1L knockdown and c-Myc restoration. Gray ratio of each blot was analyzed by using the Image J software and protein/GAPDH ratio was shown. **f, g** The effect of c-Myc restoration on tumorigenicity of SW480 and HCT116 cells after DOT1L knockdown. Tumor volume, tumor weight, and H&E staining were performed to determine the capacity of tumorigenicity

To further verify the relationship between DOT1L and c-Myc, we overexpressed c-Myc in SW480 and HCT116 cells with DOT1L silencing (Fig. 8e). According to the MTT, BrdU, and soft agar experiments, we found that the cell proliferation, capability of colony formation, as well as tumorigenicity were recovered to a certain extent in c-Myc-restorated SW480 and HCT116 cells after DOT1L silencing, compared with cells with DOT1L silencing (Fig. 8a–c). Besides, the cell cycle arrest and the dysregulated expression of cell cycle–related proteins were also rescued to a certain extent, in c-Myc-restorated SW480 and HCT116 cells after DOT1L silencing, compared with cells with DOT1L silencing (Fig. 8d, e). Importantly, c-Myc restoration also recovered the suppression of HCT116 xenografts in nude mice induced by DOT1L silencing (Fig. 8f, g). These evidences suggested that DOT1L/c-Myc axis plays an essential role in CRC progression both in vitro and in vivo.

## Discussion

CRC is the third most common cancer and the third leading cause of cancer death in men and women in the USA [58]. While in China, the CRC was the fourth most common cancer and the fifth leading cause of cancer death in China [59]. There are lower rates of incidence (14.2 per 100,000), mortality (7.4 per 100,000), and 5-year prevalence (52.7 per 100,000) of CRC in China than that in the developed countries [60]. Besides, the incidence and mortality are increasing during the last decades. It is important to have a better understanding of the nature of CRC, thus may contribute to exploring new therapeutic strategies for the treatment of this disease.

Epigenetic dysregulations are important in the modulation of tumorigenesis and cancer development [61], and also play emerging roles in the initiation and progression of CRC [62–65]. Dysregulated epigenetics in CRC particularly regard aberrant DNA methylation, alterations in histone modification states, and noncoding RNA deregulation [66]. In addition, these epigenetic alterations are promising biomarkers for diagnostic, prognostic, and therapeutic applications [66]. Recently, abnormal histone modifications, including histone methylation, acetylation, and phosphorylation, as well as other post-transcriptional modifications, such as ubiquitination, palmitoylation, myristoylation, and ADP-ribosylation are demonstrated to be essential in CRC initiation, progression, and metastasis [65, 67]. H3K79 methylation is also important for understanding the etiology of tumors [11]. However, the detailed correlations of H3K79 methylation underlying in CRC remain unclear.

Some researchers reported that H3K79 methylation in CRC was not significantly higher than that in normal tissues [68]. However, in this study, we found that DOT1L, the only identified H3K79 methyltransferase [69], was highly expressed in CRC tissues and correlated well with the prognosis of patients with colon cancers. Besides, DOT1L silencing or inhibition induced cell proliferation suppression in vitro and tumorigenicity in vivo in human CRC cells. These evidences indicated that DOT1L might be a promising prognostic or therapeutic target in CRC.

Interestingly, DOT1L silencing or inhibition by its specific inhibitor EPZ004777 induced significant decline of H3K79 methylation, including mono-, di-, and tri-methylations. As a proto-oncogene, c-Myc, is frequently overexpressed in up to 70–80% of colon adenocarcinomas [70] and is also an important therapeutic target for CRC treatment [71]. As a basic-helix-loop-helix-leucine zipper protein, c-Myc can occupy regulatory regions of up to 15% of all genes, thereby activating or repressing its expression, thus involving cell proliferation, transformation, cell cycle, metabolism, metastasis, and apoptosis [72, 73]. We identified that DOT1L expression was positively correlated with MYC expression in several cohorts of patients with CRC, and silencing or inhibition also reduced the expression of c-Myc in both mRNA or protein levels both in vitro and in vivo. Importantly, H3K79me2 was enriched on the − 6682~+ 284 region of c-Myc promoter in both SW480 and HCT116 cells. Actually, a recent study also reported that DOT1L could transcriptionally activate c-Myc expression in multiple myeloma [74]. Our data showed the detailed mechanism of epigenetic regulation of c-Myc in CRC cells. Besides, DOT1L can also form a complex with c-Myc and p300 to control the expression of EMT-related transcriptional factors and promote EMT-induced cancer stem cell properties in human breast cancer [22]. We also found that DOT1L can form a complex with c-Myc in CRC cells (data not shown). Importantly, we found that DOT1L high expression was correlated with CRC metastasis (Additional file 1: Figure S3I–L), which was tightly related to EMT. But the exact relationship of DOT1L and EMT needs to be further studied.

Besides, DOT1L silencing or inhibition induced significant cell cycle arrest at S phase, as well as the decreased protein and mRNA expression of regulators (CDK2, Cyclin A2) in the S phase checkpoint. Moreover, proliferating cell nuclear antigen (PCNA), which acted as an auxiliary protein for DNA polymerase-δ and helped increase the processivity of leading strand synthesis during DNA replication [75], was also declined by DOT1L silencing or inhibition (Fig. 4e). It was reported that the expression of c-Myc and PCNA were both highly expressed in metastasizing human CRC [76]. On the other hand, p21^WAF1/CIP1^ and p27, two important CDKs (including CDK2) inhibitors, which were transcriptionally inhibited by c-Myc [77, 78], directly interacted with PCNA, and blocks DNA replication [79, 80]. In addition, Cyclin A2 interplayed with CDK2 to control the G1/S transition, or interplayed with CDK1 and CDK2 to control G2/M entry [81]. Besides, Cyclin A2 also could be induced by c-Myc, thereby promoting cell cycle in mammary cancer cells [82]. In our study, we found that restoration of c-Myc rescued the dysregulated expression of p21, p27, CDK2, and Cyclin A2 expression, as well as cell cycle arrest in both SW480 and HCT116 cells. These results implied that DOT1L contributes to cell cycle progression probably through c-Myc-controlled p21, p27, CDK2, PCNA, and Cyclin A2 expression.

The histone methyltransferases DOT1L and EZH2, as well as the demethylase LSD1, are three promising histone methyltransferases and demethylases in clinical trials for cancer therapy [83]. Previously, EPZ00477 showed a good effect on the treatment of mixed lineage leukemia [32] and DNMT3A-mutant acute myeloid leukemia [84]. Importantly, we also found that EPZ004777 inhibits cell proliferation and growth of CRC cells both in vitro and in vivo, without affecting the weight of mice. Our data provided the primary data about the anticancer effect of EPZ004777 in solid tumors, especially CRC.

## Conclusion

In summary, our data provide the evidences that epigenetic downregulation of c-Myc by DOT1L silencing or inhibition induces significant cell proliferation suppression, cell cycle arrest at S phase, and inhibits tumor growth in vivo. Besides, we also showed that EPZ004777 has the potentiality to be a promising drug for CRC treatment. Mechanic studies showed that DOT1L enriches on the promoter of c-Myc and promotes its transcription, thereby inducing the expression of CDK2, Cyclin A2, as well as PCNA expression, which contributes to cell cycle progression. In conclusion, we indicated that DOT1L might be prognostic and therapeutic biomarkers in CRC, and EPZ004777 might be a promising drug for CRC treatment.

## Supplementary information

**Additional file 1: Figure S1.** DOT1L DNA copy number in colorectal cancer cell lines is higher than that of other types of tumor cell lines. Data was analyzed in the Garnett Cellline datasheet in the Oncomine platform. **Figure S2.** DOT1L is highly expressed in colorectal cancer. **a** Relative mRNA expression of DOT1L in colon carcinoma cells or carcinoma-associated fibroblasts in Carmical datasheet from the R2 platform. **b** DNA copy number of DOT1L in ascending colon, descending colon, rectum, COAD or rectum adenocarcinoma (READ) tissues in Kurashina Colon datasheet from the Oncomine. COAD and READ were analyzed independently in the statistical analysis by using ANOVA. **c** Relative mRNA expression of DOT1L in COAD, colorectal mucinous adenocarcinoma, READ or rectosigmoid adenocarcinoma tissues in the TCGA datasheet from the Oncomine. **d** The DNA copy number of DOT1L in different subgroups of colorectal cancers. **e** Relative mRNA expression of DOT1L in distal or proximal colon cancer tissues in Marisa datasheet from the R2 platform. **Figure S3.** DOT1L is highly expressed in high-risk colorectal cancer and predicts lower prognosis. **a-f** DOT1L mRNA expression in colon adenocarcinoma with microsatellites stability (MSS) or microsatellites stability (MSI) in different datasheets from the R2 platform. **g** DOT1L mRNA expression in colon adenocarcinoma with Braf mutation (MT) or not (wild type, WT) in Wessels cohorts from the R2 platform. **h** DOT1L mRNA expression in COAD specimens with or without node tumor deposits in the TCGA COAD datasheet from the R2 platform. **i** DOT1L mRNA expression in COAD specimens with or without lymph nodes examined count in the TCGA COAD datasheet from the R2 platform. **j** DOT1L mRNA expression in primary or metastatic colon cancer specimens in Yagi Colon FOLFOX datasheet from the R2 platform. **k** DOT1L mRNA expression in normal colon, primary tumor or liver/lung metastatic colon cancer specimens in Domany Colon datasheet from the R2 platform. **l** DOT1L mRNA expression in colon cancer specimens from patients with different levels of Metastatic spinal cord compression (MSCC) in Clary Colon datasheet from the R2 platform. **m** DOT1L mRNA expression in colon cancer specimens from patients with or without responder to FOLFOX6 treatment in Yagi Colon FOLFOX datasheet from the R2 platform. **n**-**p** DOT1L mRNA expression in colon adenocarcinoma from patients with different genders in 3 different cohorts.DOT1L mRNA expression in colon cancer specimens from male or female patients in Wessels Colon datasheet from the R2 platform. **q** DOT1L mRNA expression in COAD specimens from patients with different races in the TCGA COAD datasheet from the R2 platform. **r** Kaplan-Meire analysis of the relationship of DOT1L expression with relapse-free survival (RFS) probability in MVRM SieberSmith Colon cancer cohorts from the R2 platform. **Figure S4.** DOT1L expression in several colorectal cancer cell lines. **a** Relative mRNA expression of DOT1L in several colorectal cancer cell lines was detected by using qRT-PCR. **b** Protein expression of DOT1L in several colorectal cancer cell lines was detected by Western blot. **c** and **d** SW480 cells was treated with different concentrations of EPZ004777 for 48 h and then DOT1L mRNA and protein expression were analyzed by using qRT-PCR and Western blot. Grey ration of each blot was analyzed by using the Image J software and DOT1L/GAPDH ratio was shown. n.s.=no sense. **Figure S5.** The correlation between DOT1L and c-Myc expression in patients with colorectal cancer. The relative expression data were analyzed in two different cohorts: **a** Tumor Colon-Smith-232-MAS5.0-u133p2 from R2 platform and **b** TCGA COAD Tumor+GTEx databases from GEPIA platform. **c** CHIP-seq data (GSE74812; BED files) of H3K79me2 and H3K79me3 in human t(4;11) cell line was downloaded from GEO and analyzed by using the IGV 2.6.3 software.

## Data Availability

The datasets used and/or analyzed during the current study are available from the corresponding author on reasonable request.

## Abbreviations

ADP: Adenosine diphosphate
ANOVA: One-way analysis of variance
BrdU: Bromodeoxyuridine (5-bromo-2′-deoxyuridine)
CDK2: Cyclin-dependent kinase 2
ChIP: Chromatin immunoprecipitation
Cimp−: :Low CpG island methylator phenotype
Cimp+: High CpG island methylator phenotype
C-Myc: MYC proto-oncogene, BHLH transcription factor
COAD: Colon adenocarcinoma
CRC: Colorectal cancer
CSC: Cancer stem cell
DFS: Disease-free survival
DMEM: Dulbecco’s modified Eagle’s medium
dMMR: Mismatch repair-deficient
DMSO: Dimethyl sulfoxide
DOT1L: Disruptor of telomeric silencing-1-like
ERα: Estrogen receptor α
GEPIA: Gene Expressing Profiling Interactive Analysis
GFP: Green fluorescent protein
H&E: Hematoxylin-eosin
H3K79: Lysine 79 histone 3
HNSCC: head and neck squamous cell carcinoma
IHC: Immunohistochemistry
KM: Kaplan-Meier
MLL: Mix lineage leukemia
MSI: Microsatellites instability
MSS: Microsatellites stability
MTT: 3-(4,5-dimethylthiazol-2-yl)-2,5-diphenyltetrazolium bromide
OS: Overall survive
PCNA: Proliferating cell nuclear antigen
PI: Propidium iodide
pMMR: Mismatch repair-proficient
READ: Rectum adenocarcinoma
RFS: Relapse-free survival
RPMI-1640: Roswell Park Memorial Institute-1640
RT-PCR: Reverse transcription polymerase chain reaction
SAM: S-adenosyl-L-methionine
SET: Su(var)3-9, enhancer-of-zeste and trithorax
TCGA: The Cancer Genome Atlas
