# Correction to: Silencing or inhibition of H3K79 methyltransferase DOT1L induces cell cycle arrest by epigenetically modulating c-Myc expression in colorectal cancer

**DOI:** 10.1186/s13148-022-01288-6

**Published:** 2022-05-21

**Authors:** Liqun Yang, Qian Lei, Lin Li, Jie Yang, Zhen Dong, Hongjuan Cui

**Affiliations:** 1 State Key Laboratory of Silkworm Genome Biology, Institute of Sericulture and Systems Biology, Southwest University, No.2, Tiansheng Road, Beibei, Chongqing, 400716 China; 2 Cancer Center, Medical Research Institute, Southwest University, Beibei, Chongqing, 400716 China; 3 Engineering Research Center for Cancer Biomedical and Translational Medicine, Southwest University, Beibei, Chongqing, 400716 China; 4 Chongqing Engineering and Technology Research Center for Silk Biomaterials and Regenerative Medicine, Southwest University, Beibei, Chongqing, 400716 China

## Correction to: Clinical Epigenetics (2019) 11: 199 10.1186/s13148-019-0778-y

Following the publication of the original article [1], the authors noted that images of Figs. 7I and 8C were misplaced by mistake. The corrected Figs. 7I and 8C are now shown in this correction. The authors confirm that the conclusions of this paper are not affected, and sincerely apologized for the mistake and any inconvenience that may have caused. The correct figures are shown in Figs. 7 and 8.

**Fig. 7:**
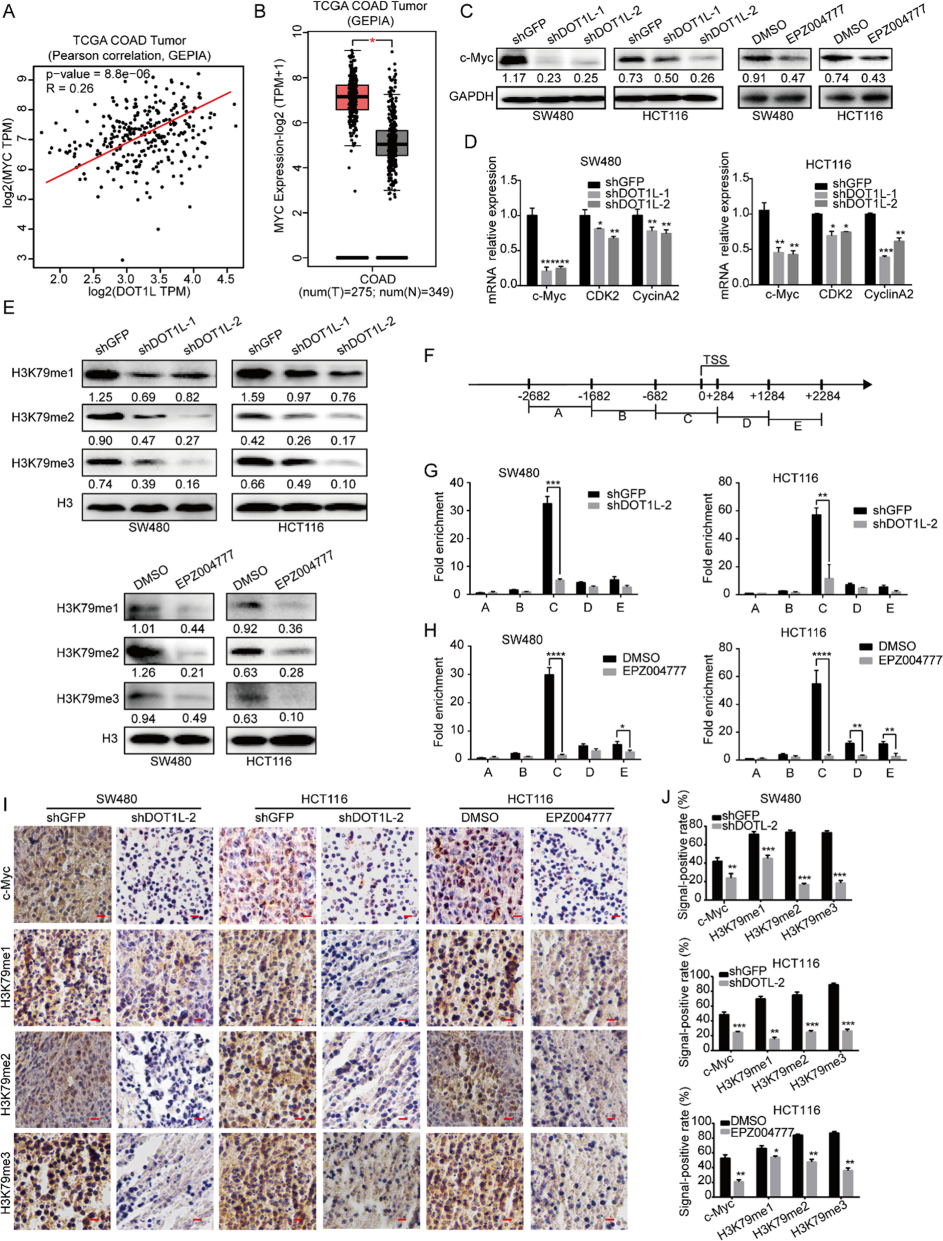
DOT1L silencing or inhibition demethylates H3K79 and suppresses transcription of c-Myc. **a** The Pearson correlation between DOT1L and c-Myc expression in patients with colorectal cancer in the TCGA COAd datasheet from the GEPIA. **b** Relative mRNA expression of c-Myc in patients with colorectal cancer in the TCGA COAd datasheet from the GEPIA. **c** Protein expression of c-Myc in SW480 and HCT116 cells after DOT1L knockdown or inhibition. Gray ratio of each blot was analyzed by using the ImageJ software and protein/GAPDH ratio was shown. **d** mRNA expression of c-Myc, CDK2, Cyclin A2 in SW480, and HCT116 cells after DOT1L knockdown or inhibition. **e** H3K9 methylation (m1/2/3) was detected by using Western blot in SW480 and HCT116 cells after DOT1L knockdown or inhibition. Gray ratio of each blot was analyzed by using the ImageJ software and protein/H3 ratio was shown. **f–h** ChIP assay was performed to detect the binding region of H3K79me2 on the promoter of c-Myc in SW480 and HCT116 cells after DOT1L knockdown or inhibition. **i**, **j** IHC staining of c-Myc and H3K79me1/2/3 in xenografts obtained from subcutaneous injecting SW480 and HCT116 cells after DOT1L knockdown or inhibition within mice. Signal-positive rate was analyzed by using the IHC profiler in the ImageJ software

**Fig. 8:**
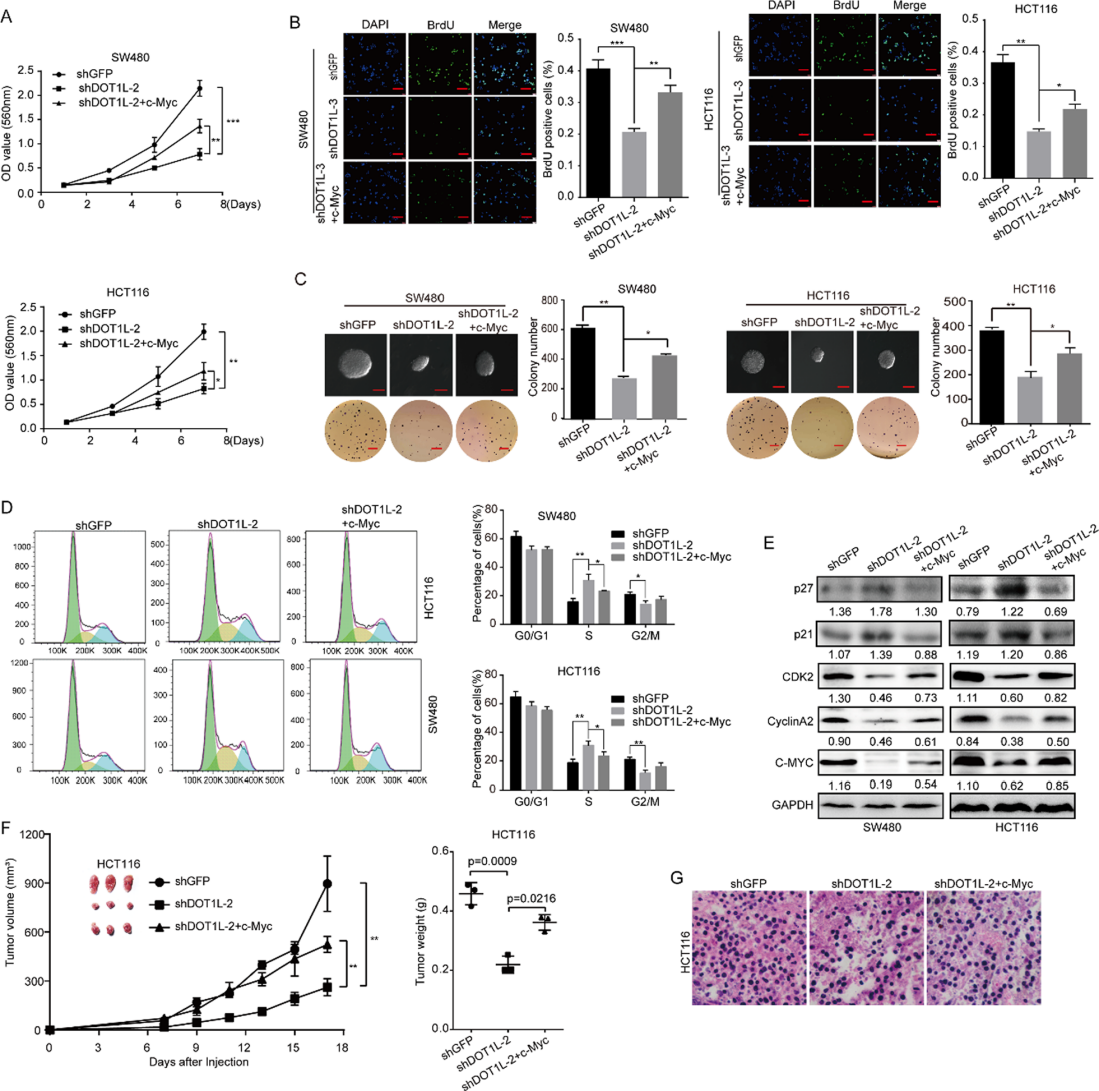
Restoration of c-Myc partly rescued cell proliferation inhibition and cell cycle arrest induced by DOT1L silencing or inhibition in vitro and in vivo. **a** Cell growth curve was determined by using MTT assay in SW480 and HCT116 cells after DOT1L knockdown and c-Myc restoration. Vector control for c-Myc overexpression was used in both shGFP and shDOT1L-2 groups (similarly hereinafter). **b** BrdU assay was performed in SW480 and HCT116 cells after DOT1L knockdown and c-Myc restoration. **c** Soft agar assay was performed in SW480 and HCT116 cells after DOT1L knockdown and c-Myc restoration. **d** Cell cycle was detected by using flow cytometry in SW480 and HCT116 cells after DOT1L knockdown and c-Myc restoration. **e** Protein expression of cell cycle-related proteins including p21, p27, CDK2, Cyclin A2, and PCNA was detected by using Western blot in SW480 and HCT116 cells after DOT1L knockdown and c-Myc restoration. Gray ratio of each blot was analyzed by using the ImageJ software and protein/GAPDH ratio was shown. **f, g** The effect of c-Myc restoration on tumorigenicity of SW480 and HCT116 cells after DOT1L knockdown. Tumor volume, tumor weight, and H&E staining were performed to determine the capacity of tumorigenicity
