# Supplementary material for: Silencing or inhibition of H3K79 methyltransferase DOT1L induces cell cycle arrest by epigenetically modulating c-Myc expression in colorectal cancer
Source: Clin Epigenetics. 2019 Dec 30;11:199. doi: 10.1186/s13148-019-0778-y (PMC6937672; doi:10.1186/s13148-019-0778-y)
Supplement: Supplementary file 1 — Additional file 1: Figure S1. DOT1L DNA copy number in colorectal cancer cell lines is higher than that of other types of tumor cell lines. Data was analyzed in the Garnett Cellline datasheet in the Oncomine platform. Figure S2. DOT1L is highly expressed in colorectal cancer. a Relative mRNA expression of DOT1L in colon carcinoma cells or carcinoma-associated fibroblasts in Carmical datasheet from the R2 platform. b DNA copy number of DOT1L in ascending colon, descending colon, rectum, COAD or rectum adenocarcinoma (READ) tissues in Kurashina Colon datasheet from the Oncomine. COAD and READ were analyzed independently in the statistical analysis by using ANOVA. c Relative mRNA expression of DOT1L in COAD, colorectal mucinous adenocarcinoma, READ or rectosigmoid adenocarcinoma tissues in the TCGA datasheet from the Oncomine. d The DNA copy number of DOT1L in different subgroups of colorectal cancers. e Relative mRNA expression of DOT1L in distal or proximal colon cancer tissues in Marisa datasheet from the R2 platform. Figure S3. DOT1L is highly expressed in high-risk colorectal cancer and predicts lower prognosis. a-f DOT1L mRNA expression in colon adenocarcinoma with microsatellites stability (MSS) or microsatellites stability (MSI) in different datasheets from the R2 platform. g DOT1L mRNA expression in colon adenocarcinoma with Braf mutation (MT) or not (wild type, WT) in Wessels cohorts from the R2 platform. h DOT1L mRNA expression in COAD specimens with or without node tumor deposits in the TCGA COAD datasheet from the R2 platform. i DOT1L mRNA expression in COAD specimens with or without lymph nodes examined count in the TCGA COAD datasheet from the R2 platform. j DOT1L mRNA expression in primary or metastatic colon cancer specimens in Yagi Colon FOLFOX datasheet from the R2 platform. k DOT1L mRNA expression in normal colon, primary tumor or liver/lung metastatic colon cancer specimens in Domany Colon datasheet from the R2 platform. l DOT1L mRNA expression in [file 13148_2019_778_MOESM1_ESM.docx]

# Supplementary Figures


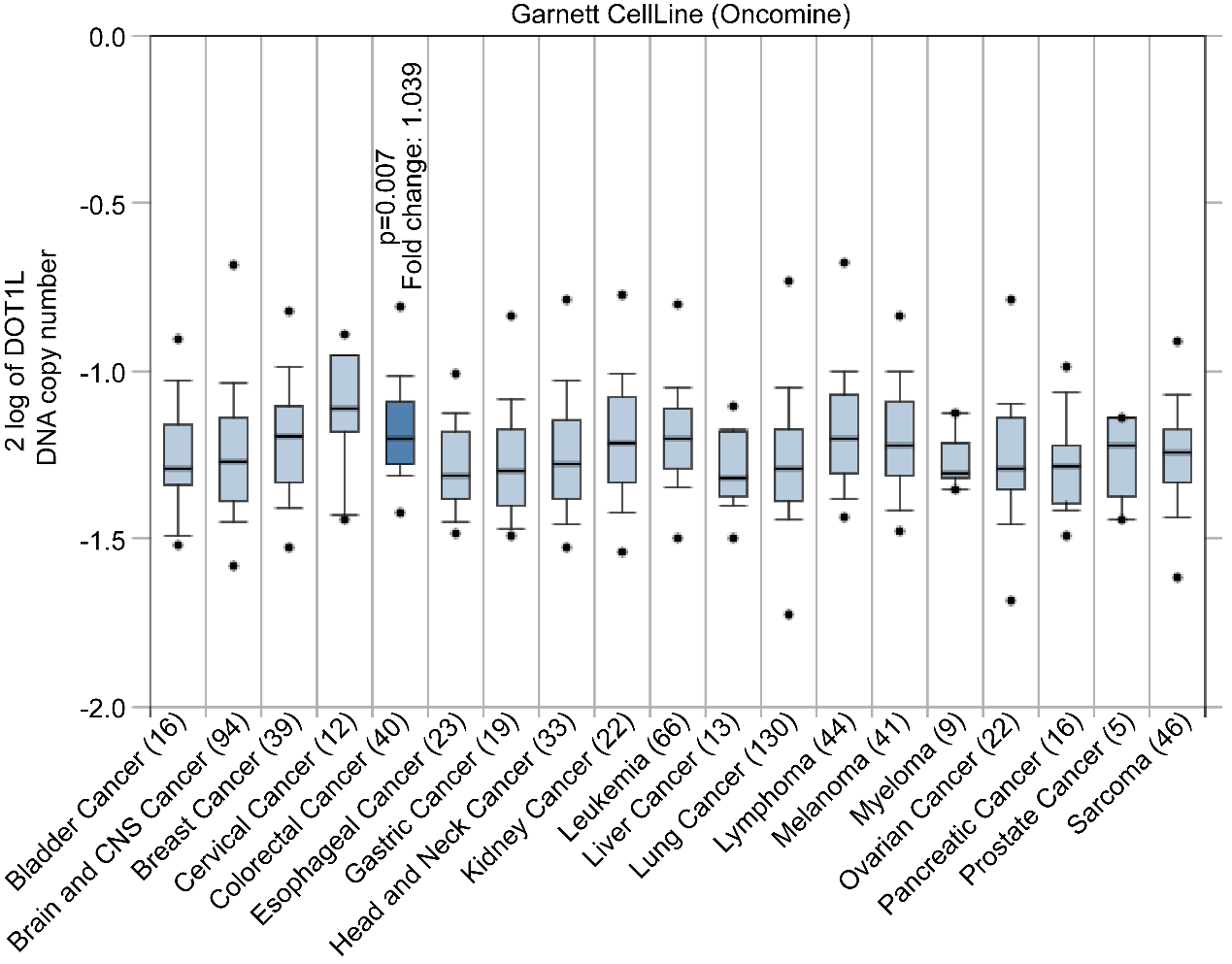


**Fig. S1** DOT1L DNA copy number in colorectal cancer cell lines is higher than that of other types of tumor cell lines. Data was analyzed in the Garnett Cellline datasheet in the Oncomine platform.


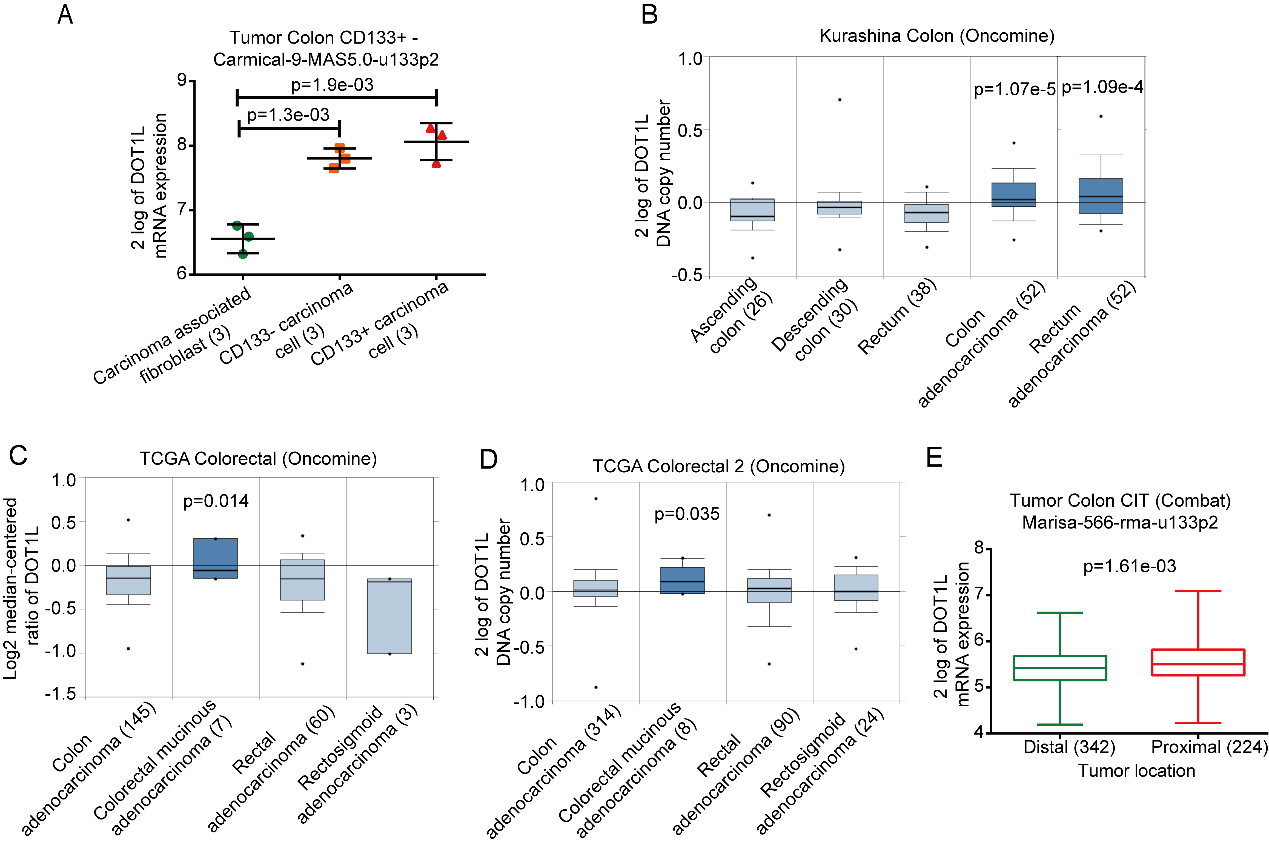


**Fig. S2** DOT1L is highly expressed in colorectal cancer. **a** Relative mRNA expression of DOT1L in colon carcinoma cells or carcinoma-associated fibroblasts in Carmical datasheet from the R2 platform. **b** DNA copy number of DOT1L in ascending colon, descending colon, rectum, COAD or rectum adenocarcinoma (READ) tissues in Kurashina Colon datasheet from the Oncomine. COAD and READ were analyzed independently in the statistical analysis by using ANOVA. **c** Relative mRNA expression of DOT1L in COAD, colorectal mucinous adenocarcinoma, READ or rectosigmoid adenocarcinoma tissues in the TCGA datasheet from the Oncomine. **d** The DNA copy number of DOT1L in different subgroups of colorectal cancers. **e** Relative mRNA expression of DOT1L in distal or proximal colon cancer tissues in Marisa datasheet from the R2 platform.


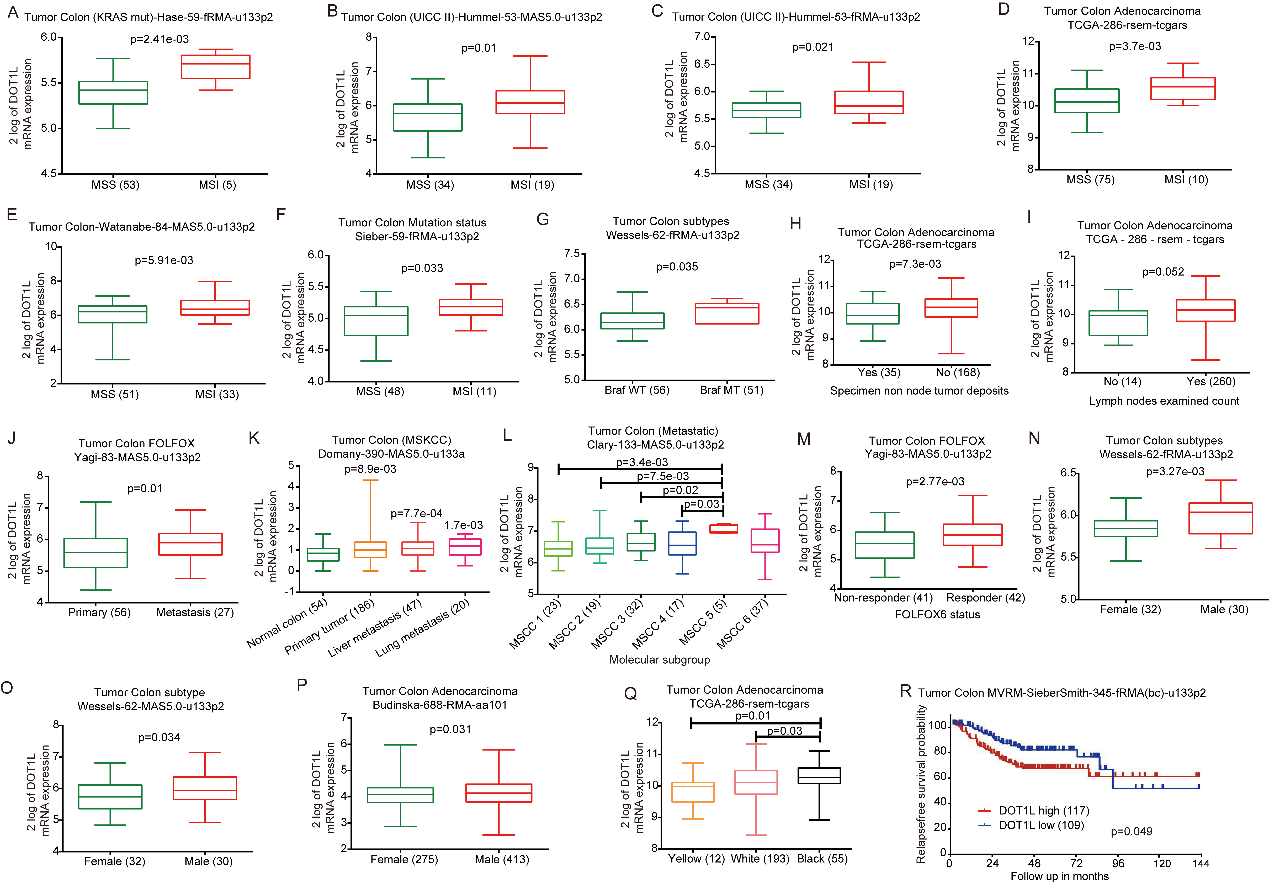


**Fig. S3** DOT1L is highly expressed in high-risk colorectal cancer and predicts lower prognosis. **a-f** DOT1L mRNA expression in colon adenocarcinoma with microsatellites stability (MSS) or microsatellites stability (MSI) in different datasheets from the R2 platform. **g** DOT1L mRNA expression in colon adenocarcinoma with Braf mutation (MT) or not (wild type, WT) in Wessels cohorts from the R2 platform. **h** DOT1L mRNA expression in COAD specimens with or without node tumor deposits in the TCGA COAD datasheet from the R2 platform. **i** DOT1L mRNA expression in COAD specimens with or without lymph nodes examined count in the TCGA COAD datasheet from the R2 platform. **j** DOT1L mRNA expression in primary or metastatic colon cancer specimens in Yagi Colon FOLFOX datasheet from the R2 platform. **k** DOT1L mRNA expression in normal colon, primary tumor or liver/lung metastatic colon cancer specimens in Domany Colon datasheet from the R2 platform. **l** DOT1L mRNA expression in colon cancer specimens from patients with different levels of Metastatic spinal cord compression (MSCC) in Clary Colon datasheet from the R2 platform. **m** DOT1L mRNA expression in colon cancer specimens from patients with or without responder to FOLFOX6 treatment in Yagi Colon FOLFOX datasheet from the R2 platform. **n**-**p** DOT1L mRNA expression in colon adenocarcinoma from patients with different genders in 3 different cohorts.DOT1L mRNA expression in colon cancer specimens from male or female patients in Wessels Colon datasheet from the R2 platform. **q** DOT1L mRNA expression in COAD specimens from patients with different races in the TCGA COAD datasheet from the R2 platform. **r** Kaplan-Meire analysis of the relationship of DOT1L expression with relapse-free survival (RFS) probability in MVRM SieberSmith Colon cancer corhorts from the R2 platform.


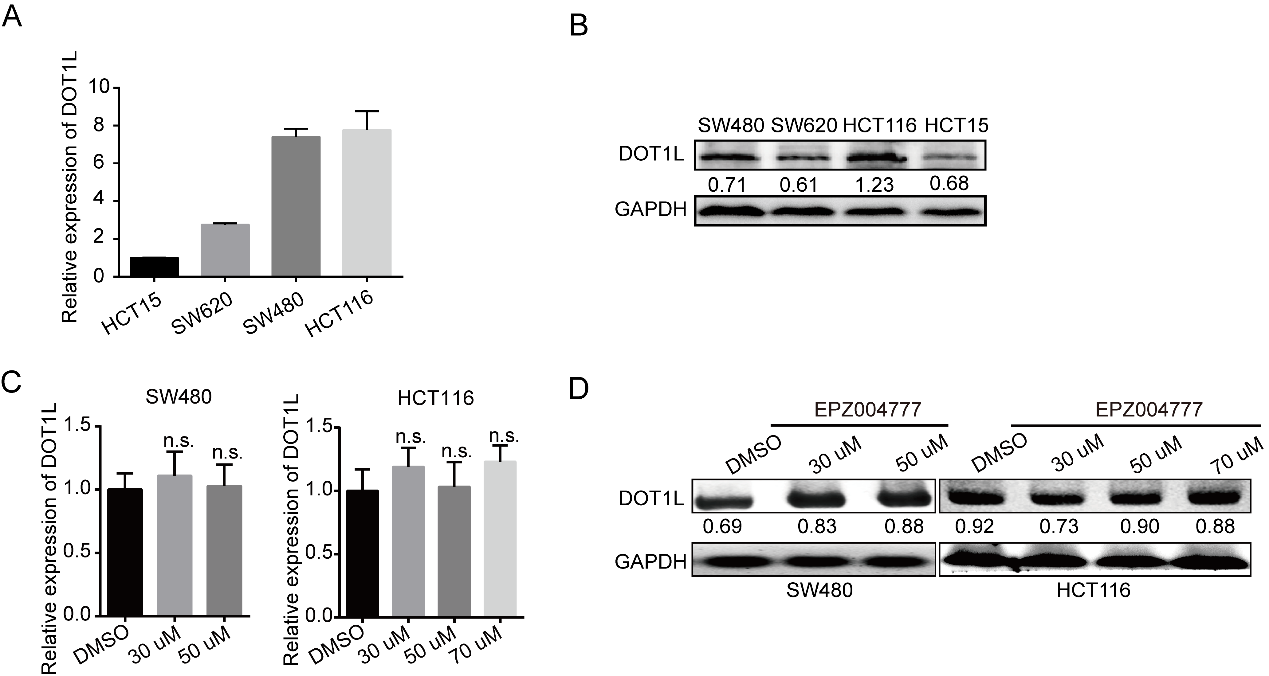


**Fig. S4** DOT1L expression in several colorectal cancer cell lines. **a** Relative mRNA expression of DOT1L in several colorectal cancer cell lines was detected by using qRT-PCR. **b** Protein expression of DOT1L in several colorectal cancer cell lines was detected by Western blot. **c** and **d** SW480 cells was treated with different concentrations of EPZ004777 for 48 h and then DOT1L mRNA and protein expression were analyzed by using qRT-PCR and Western blot. Grey ration of each blot was analyzed by using the Image J software and DOT1L/GAPDH ratio was shown. n.s.=no sense.


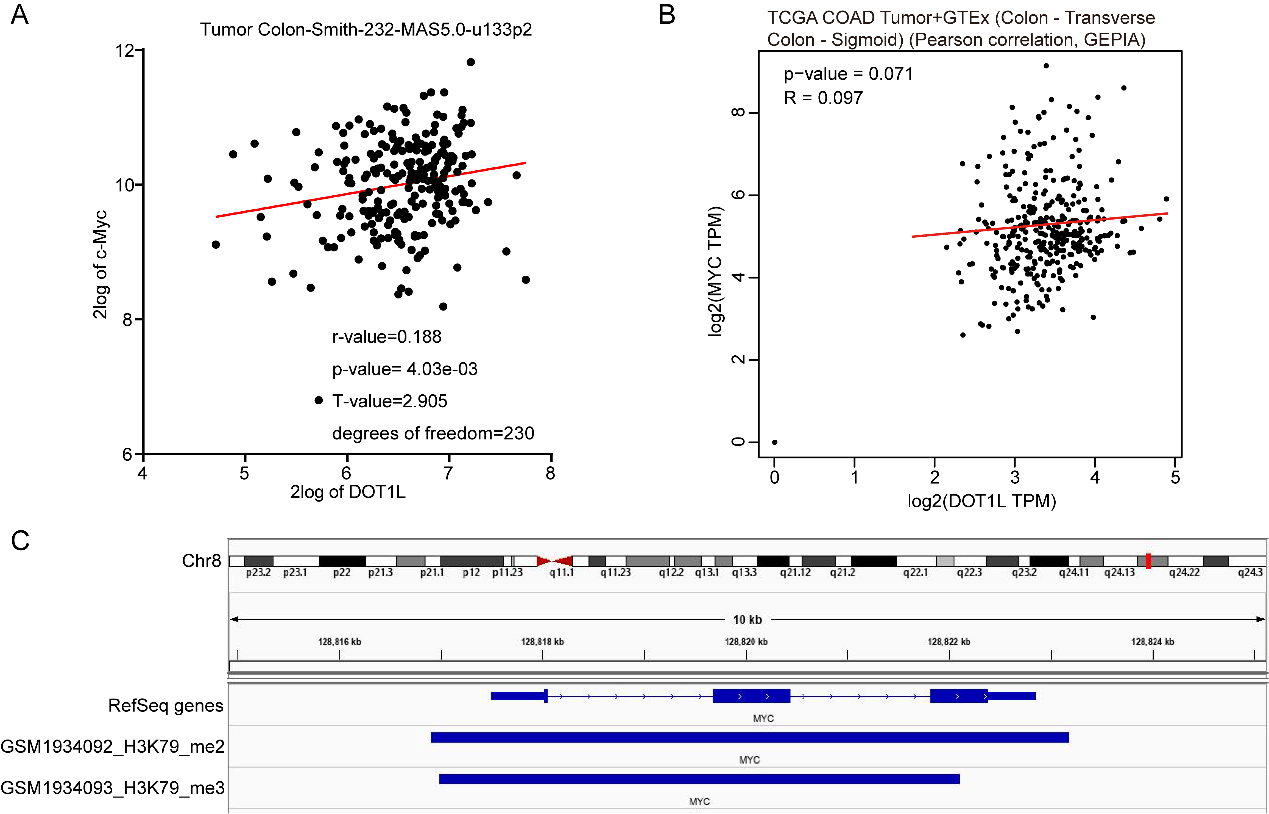


**Fig. S5** The correlation between DOT1L and c-Myc expression in patients with colorectal cancer. The relative expression data were analyzed in two different cohorts: **a** Tumor Colon-Smith-232-MAS5.0-u133p2 from R2 platform and **b** TCGA COAD Tumor+GTEx databases from GEPIA platform. **c** CHIP-seq data (GSE74812; BED files) of H3K79me2 and H3K79me3 in human t(4;11) cell line was downloaded from GEO and analyzed by using the IGV 2.6.3 software.
